# Anti-leukemia activity of the ethyl acetate extract from *Gynostemma pentaphyllum* (Thunb.) leaf against FLT3-overexpressing AML cells and its phytochemical characterization

**DOI:** 10.1186/s12906-025-04903-0

**Published:** 2025-05-13

**Authors:** Khin Khin Gyi, Songyot Anuchapreeda, Nutjeera Intasai, Montree Tungjai, Siriporn Okonogi, Arihiro Iwasaki, Toyonobu Usuki, Singkome Tima

**Affiliations:** 1 Department of Medical Technology, Faculty of Associated Medical Sciences, Chiang Mai University, Chiang Mai, 50200 Thailand; 2 Faculty of Associated Medical Sciences, Chiang Mai University, Under The CMU Presidential Scholarship, Chiang Mai, 50200 Thailand; 3 Department of Materials and Life Sciences, Faculty of Science and Technology, Sophia University, 7-1 Kioicho, Chiyoda-ku, Tokyo, 102-8554 Japan; 4 Cancer Research Unit of Associated Medical Sciences (AMS CRU), Faculty of Associated Medical Sciences, Chiang Mai University, Chiang Mai, 50200 Thailand; 5 Center of Excellence in Pharmaceutical Nanotechnology, Chiang Mai University, Chiang Mai, 50200 Thailand; 6 Department of Radiologic Technology, Faculty of Associated Medical Sciences, Chiang Mai University, Chiang Mai, 50200 Thailand; 7 Department of Pharmaceutical Sciences, Faculty of Pharmacy, Chiang Mai University, Chiang Mai, 50200 Thailand; 8 Department of Applied Chemistry, Faculty of Science and Engineering, Chuo University, 1-13-27 Kasuga, Bunkyo-ku, Tokyo, 112-8551 Japan

**Keywords:** Acute myeloid leukemia, *Gynostemma pentaphyllum* (Thunb.), FLT3, WT1, Anti-leukemia, Dehydrovomifoliol

## Abstract

**Background:**

*Gynostemma pentaphyllum* (Thunb.), a traditional adaptogenic herb, is known for its bioactive components with potential anti-cancer properties. Acute myeloid leukemia (AML) progression is significantly influenced by Feline McDonough Sarcoma (FMS)-like tyrosine kinase 3 (FLT3) signaling, while Wilms’ tumor 1 (WT1) serves as a key prognostic marker. This study investigates the anti-leukemia activities of active *G. pentaphyllum* leaf extracts and their components, focusing on the inhibition of FLT3 and WT1 activity.

**Methods:**

*G. pentaphyllum* extracts were prepared through maceration, yielding three crude fractional extracts. The cytotoxicity of the extracts was screened against various leukemia cell lines using the 3-(4,5-dimethylthiazol-2-yl)-2,5-diphenyl tetrazolium bromide (MTT) assay. The most cytotoxic extract was further fractionated and purified via column chromatography. The anti-proliferative and apoptotic induction activities of the active extract and its fraction were evaluated through cell cycle and apoptosis analyses using flow cytometry. Changes in mitochondrial membrane potential (ΔΨm) were assessed by spectrofluorometry. To confirm anti-leukemia activity, the expression levels of FLT3, WT1 and apoptotic-related protein were analyzed using Western blotting. The major active compounds within the active fractions were identified and characterized using Electrospray Ionization Mass Spectrometry (ESI-MS) and Nuclear Magnetic Resonance (NMR) spectroscopy.

**Results:**

The ethyl acetate fractional extract (F-EtOAc) demonstrated the highest cytotoxicity, particularly against FLT3-overexpressing EoL-1 (IC_50_ = 40.82 ± 0.8 µg/mL) and MV4-11 (IC_50_ = 35.54 ± 4.1 µg/mL) AML cell lines. Fraction F10 was identified as the most active fraction, significantly inhibited FLT3 and WT1 protein expression and induced G0/G1 cell cycle arrest in a dose-dependent manner. Additionally, F10 induced dose-dependent apoptosis through disruption of ΔΨm, p53 up-regulation and caspase-3 activation. Further purification of F10 identified dehydrovomifoliol as its major bioactive compound.

**Conclusion:**

These findings suggest that the ethyl acetate extract of *G. pentaphyllum* contains bioactive compounds with anti-leukemia potential, warranting further investigation to evaluate its efficacy against AML.

**Clinical trial number:**

Not applicable.

**Supplementary Information:**

The online version contains supplementary material available at 10.1186/s12906-025-04903-0.

## Introduction

Cancer remains a leading global cause of mortality despite extensive research efforts. Leukemia, characterized by transformed malignant hematopoietic progenitors and bone marrow infiltration, poses a significant health burden, particularly in countries with middle to high sociodemographic indices (SDI) [1]. In 2022, leukemia ranked as the 13th most frequently diagnosed cancer and the 10th leading cause of cancer-related mortality globally [2]. In Thailand, it was among the top 15 cancers, being the 11th most common cancer and the 9th leading cause of cancer-related mortality in 2020 [3]. AML is a heterogeneous clonal disorder marked by uncontrolled cell proliferation and impaired differentiation of immature myeloid blasts. AML accounts for 33% of all blood cancers and is characterized by the lowest survival rates, with a 5-year survival rate of 24% [4]. Aberrant expression of the FLT3 receptor protein on the surface of AML cells is linked to disease pathogenesis and poor prognosis [5]. FLT3’s pivotal role in AML cell proliferation and survival, along with its frequent mutation and overexpression in a substantial proportion of AML patients [5, 6], positions FLT3 as an appealing therapeutic target. The WT1 nuclear protein, encoded by the *WT1* gene containing four zinc fingers, serves as a transcriptional activator or repressor [7]. WT1 is predominantly expressed in progenitor cells, but at lower levels in mature normal hematopoietic cells [7]. Numerous studies have highlighted the potential oncogenic role of WT1 in leukemia progression, with its expression serving as a marker for leukemic cell proliferation [8]. Given the significant roles of FLT3 and WT1 in leukemia pathogenesis, ongoing research to identify natural bioactive compounds targeting these pathways is essential, as it offers the potential for effective therapies with minimal side effects.

Inducing apoptosis in cancer cells is a desirable treatment outcome, with mitochondria playing a crucial role in regulating the initiation and execution of this process. At the molecular level, both extrinsic and intrinsic apoptotic pathways are activated in response to appropriate stimuli, such as granzyme and perforin, along with the regulatory caspase cascade [9]. Genetically, p53, a 53-kDa nuclear phospho-protein, binds to DNA and functions as a transcription factor that controls cell proliferation and DNA repair [10]. Activation of p53 in cell culture and animal experiments leads to cell-cycle arrest and apoptosis [10]. The intrinsic apoptotic pathway involves the permeabilization of the outer mitochondrial membrane, resulting in the loss of membrane potential, and the release of cytochrome c. This promotes apoptotic protease activating factor-1 (Apaf-1) oligomerization, subsequently activating effector caspases 3 and 7, ultimately inducing cell death [10, 11]. According to Yamaguchi et al., natural compounds capable of inducing apoptosis hold potential for anti-cancer drug development [12]. Similarly, any compound that arrests cell cycle progression is considered a potential mechanism to inhibit cancer cell growth [12, 13].

*Gynostemma pentaphyllum* (Thunb.), commonly known as ‘Jiaogulan’ or ‘Cheap Ginseng’, is a perennial herbaceous climbing vine belonging to the Cucurbitaceae family [14, 15]. It is the most extensively studied species within the genus *Gynostemma* [16]. For centuries in Asia, its leaves have been used in Traditional Chinese Medicine (TCM) to treat various ailments including common colds, cardiovascular diseases, hepatitis, and hyperlipidemia [17–19]. *G. pentaphyllum* leaves are rich in biologically active components and are widely prepared as dietary supplements and herbal tea, marketed in many Asian countries and the United States. These preparations are commonly used to protect against and improve conditions such as diabetes, oxidative stress, and immune function [20–22]. Several phytochemical studies have reported that highly polar glycosides, including saponins and flavonoids, as well as polysaccharides, are major active constituents of *G. pentaphyllum*, contributing to its diverse pharmacological effects [15–17]. Among these, gypenosides, a class of dammarane glycoside saponins derived from hydrolysates, ethyl acetate, or n-butanol extracts, have demonstrated efficacy against various cancer cell lines, such as human prostate cancer [23, 24], breast cancer [25], lung cancer [26], and leukemia [14, 27]. Flavonoids from *G. pentaphyllum* exhibit significant physiological activities, including the reduction of obesity risk [16] and anti-proliferative effects [28]. Additionally, crude polysaccharide extracts have shown immunomodulatory and antioxidant properties by enhancing the levels of glutathione peroxidase (GSH-Px) and superoxide dismutase (SOD) enzymes, as well as cytokines, including nitric oxide (NO), interleukin-1β (IL-1β) and tumor necrosis factor-α (TNF-α) [29, 30]. Despite these findings, the anti-leukemia mechanisms of *G. pentaphyllum* in relation to the leukemia cell proliferation markers FLT3 and WT1 remain unexplored. This study aims to investigate the anti-leukemia activities of *G. pentaphyllum* ethyl acetate extract on EoL-1 cells expressing human wild-type FLT3 (FLT3-WT) and MV4-11 cells harboring internal tandem duplication mutants of FLT3 (FLT3-ITD). Fractionation and purification were performed to isolate and identify the main active compound in the active extract and fraction. Normal human PBMCs were used to determine whether the active extract and fraction exhibit selective cytotoxicity against leukemia cells.

## Materials and methods

### Chemicals and reagents

The high-performance liquid chromatography (HPLC)-grade solvents, including methanol, chloroform, and formic acid were procured from Lab-Scan Co. (Gliwice, Poland). The analytical-grade solvents, including n-hexane, ethyl acetate, ethanol, acetone, and dimethyl sulfoxide (DMSO) were purchased from Labscan (Dublin, Ireland). MTT and Folin–Ciocalteu reagents were purchased from Sigma-Aldrich (St. Louis, MO, USA). Iscove’s Modified Dulbecco’s Media (IMDM), Roswell Park Memorial Institute (RPMI)-1640, penicillin-streptomycin, L-glutamine, and trypan blue solution (0.4%) were purchased from Gibco; Thermo FisherScientific, Inc. Fetal bovine serum (FBS) was purchased from Capricorn Scientific (Hesse, Germany). Column chromatography was performed with Silica gel 60 (40–63 μm) produced by Merck (Darmstadt, Germany). Analytical thin layer chromatography (TLC) was performed on Silica gel 60 F254 plates produced by Merck (Darmstadt, Germany).

### Collection of plant material and Preparation of *G. pentaphyllum* crude fractional extracts

Fresh leaves of *G. pentaphyllum* was a generous gift from Leticia Medina Smithapindhu, the owner of Jiaogulan (*Gynostemma pentaphyllum* (Thunb.)) Makino Plantation Chiang Mai, Thailand. The plant was harvested in May, 2021. The harvested plant material used in this study was taxonomically identified and authenticated by Miss Wannaree Charoensup, a curator botanist, at the Chiang Mai University Herbarium, Northern Research Center for Medicinal Plants, Faculty of Pharmacy, Thailand, with a voucher specimen (reference no. 0023392). *G. pentaphyllum* leaf samples (1.8 kg) were air-dried and ground into a fine powder. The crude extracts were prepared by sequential maceration in three different organic solvents, starting with n-hexane, followed by ethyl acetate and ethanol, over a 72 h period with three extraction cycles of 24 h each. After each cycle, the liquid portion was collected, pooled, and filtered through Whatman No.1 filter paper, then concentrated using a rotary evaporator (N-1000, EYELA, Shanghai, China), and subsequently dried in a hot air oven at 40 °C [31]. The extracts were then weighed and stored at -20 °C until further analyses. All crude extracts were used to evaluate their cytotoxic effect on leukemia cell models and the most active extract was selected for the next step of isolation and purification.

### Isolation and purification of the active crude extract

The isolation of active crude fractional extract was carried out by normal silica column chromatography (NSCC) using different ratios of hexane/ethyl acetate solvent system in increasing polarity. The main fractions were collected after thin-layer chromatography (TLC) confirmation. All fractions were tested for their cytotoxic effects on leukemia cell models, in which the most active fraction was selected for the next step of isolation. Multiple isolation steps were carried out using NSCC and medium-pressure liquid chromatography (MPLC) recorded on Yamazen Smart Flash EPC LC W-Prep 2XY Dual Channel Automated Flash Chromatography System, equipped with either a Yamazen Ultra Pack B silica column or a Yamazen Hi-Flash silica column. A reversed-phase high-performance liquid chromatography (RP-HPLC) system (Shimadzu, Kyoto, Japan) was employed for purification of the compound using a C_18_ column (5C_18_-AR-II, 250 mm) and a C_18_ guard column (5C_18_-MS-II, 20 mm). The column was set at 40 °C and samples (100 µL) were injected at a flow rate of 1mL/min using a binary gradient program (A = MeOH-0.1% formic acid and B = H_2_O-0.1% formic acid) to achieve optimal separation of compounds. Peaks of interest were monitored at λ 270 nm.

### Characterization of compounds

Spectroscopic methods, primarily ESI-MS and NMR, were carried out to elucidate structure of the purified compounds. ^1^H-NMR and ^13^C-NMR spectra were recorded on JEOL (Tokyo) JNM-ECA 500 spectrometer (500 MHz) after samples were dissolved in a deuterated solvent (CDCl_3_). ESI-MS spectra were recorded on JEOL JMS-T100LC instrument (Tokyo).

### Cells and culture conditions

EoL-1 (acute eosinophilic leukemia, RBRC-RCB0641), K562 (erythroleukemia), KG1 and KG1-a (acute myeloblastic leukemia with stem cell population, ATCC^®^ CCL-246™), Molt-4 (acute lymphoblastic leukemia), MV4-11 (acute biphenotypic B-myelomonocytic leukemia, ATCC^®^ CRL 9591TM), and U937 (acute monocytic leukemia) were used as human leukemic cell line models in this study. EoL-1 and MV4-11 cells demonstrated the FLT3 wild type and FLT3-ITD mutation phenotype, respectively [32]. The EoL-1, K562, Molt-4, and U937 cells were cultured in RPMI-1640 medium containing 1 mM L-glutamine, 100 units/mL penicillin, and 100 µg/mL streptomycin supplemented with 10% FBS. The MV4-11 cells were cultured in IMDM medium containing 100 units/mL penicillin, and 100 µg/mL streptomycin, supplemented with 10% FBS. The KG1 and KG1-a cells were cultured in IMDM medium containing 100 units/mL penicillin, and 100 µg/mL streptomycin, supplemented with 20% FBS. All leukemic cell lines were cultured at 37 °C in a humidified incubator with 5% CO_2_.

### Peripheral blood mononuclear cells (PBMCs) Preparation

PBMCs were isolated from the whole blood of five healthy volunteers using the Ficoll-Hypaque density gradient centrifugation. Briefly, the whole blood was mixed with an equal volume of phosphate-buffered saline (PBS, pH 7.4). Then, Ficoll-Hypaque solution (Sigma-Aldrich, St. Louis, MO, USA) was underlaid within the sample. The sample was centrifuged at 25 °C, 400 g for 30 min. Finally, the PBMCs were collected and washed three times with PBS, pH 7.4 before being used.

### Cytotoxic determination by MTT assay

The cytotoxicity of extracts and fractions were evaluated using the MTT assay. In brief, leukemic cells (1.0–3.0 × 10^4^ cells/well) and PBMCs (1.0 × 10^6^ cells/well) were seeded to each well in 96-well culture plate and incubated overnight to obtain confluency prior to treatments. Then the medium containing 0–100 µg/mL of the extracts and fractions was added. After 48 h incubation, MTT dye solution (5 mg/mL) was added and incubated at 37 °C for 4 h, followed by dissolving the formazan crystals product with 200 µL DMSO. The optical density was measured using an absorbance microplate reader (M965/ M965 + Mate 2.0, Metertech, Inc.) at 578 nm with a reference wavelength at 630 nm. The percentage of cell viability was calculated using following equation:

$$\eqalign{& \%\:Cell\,viability\, \cr & = \,{{Mean\,absorbance\,of\,test\,well} \over {Mean\,absorbance\,of\,vehicle\,control\,well}}\, \times \,100 \cr} $$

The dose-response curve was constructed using the mean percentages of cell survival for each concentration from triplicate experiments. The 50% inhibitory concentration (IC_50_) was identified as the concentration that would inhibit cell growth by 50% compared with untreated control, and the 20% inhibitory concentration (IC_20_) value was determined as non-cytotoxic dose used for the cell cycle analysis and protein expression studies.

### Total cell number of FLT3-overexpressing cells determined by the Trypan blue exclusion test

The trypan blue exclusion test was used to determine cell viability of the test samples. EoL-1 and MV4-11 cells were adjusted to 3.0 × 10^5^ and 1.5 × 10^5^ cells/mL, respectively, and incubated with three non-cytotoxic (IC_10_, IC_15_, IC_20_) concentrations of F-EtOAc and F10 at 37 °C under 5% CO_2_ atmosphere for 48 h. The treated cells were collected and re-suspended with PBS, pH 7.4. The cell suspensions were appropriately diluted with PBS, pH 7.4 before mixing with 0.2% trypan blue solution to count viable and dead cells using a hemocytometer under a light microscope. The total cell number of viable and dead cells were then calculated. Based on the concept of trypan blue exclusion, live cells with intact membranes can exclude trypan blue dye and show clear cytoplasm. In contrast, dead cells with compromised membranes can become stained by trypan blue dye solution [33].

### Analysis of cell cycle by flow cytometry

Cell cycle analysis was quantified using propidium iodide (PI), which represents the content of nuclear DNA [34]. EoL-1 (3.0 × 10^5^ cells/mL) and MV4-11 (1.5 × 10^5^ cells/mL) cells were cultured in complete RPMI-1640 and IMDM medium respectively, with three non-cytotoxic (IC_10_, IC_15_, IC_20_) concentrations of F-EtOAc and F10 for 48 h to evaluate their effect on cell cycle progression. After treatment, cells were washed twice with ice-cold PBS, pH 7.4, and prepared as single-cell suspension. Then, the cells were fixed with pre-cooled 70% ethanol for 30 min. The cell pellets were then washed twice with PBS, pH 7.4 and cells were stained for total DNA content with PI solution (0.02 mg/mL PI in PBS, 2mM EDTA, 0.1% Triton X-100, 8 µg/mL RNAse A, pH 7.4) in the dark at room temperature (25 °C). The cell cycle progression was analyzed by DxFLEX Flow Cytometer (Beckman Coulter). Results are presented as the percentage of cell population determined using FlowJo software V10 (Ashland, OR, USA).

### Analysis of apoptosis induction by flow cytometry

Cellular apoptosis was analyzed with FITC Annexin V Apoptosis Detection Kit with PI (Biolegend™, CA, USA) according to the manufacturer’s instructions. In brief, EoL-1 (3.0 × 10^5^ cells/mL) and MV4-11 (1.5 × 10^5^ cells/mL) cells were incubated with four concentrations (IC_10_, IC_20_, IC_30_, IC_50_) of F-EtOAc and F10 for 48 h. The anti-cancer drug doxorubicin (Dox, 2 mg/mL) (Fresenius Kabi Oncology, Ltd.) at the IC_50_ value was used as a positive control to set up the quadrants for cell population determination. The cells were harvested, washed with PBS, pH 7.4, and resuspended in binding buffer. Then cells were stained with Annexin V-FITC and PI for 15 min at room temperature in a dark place. At the end, more binding buffer was added and analyzed by CytoFLEX Flow Cytometer (Beckman Coulter). The percentage of cells were distributed into different populations (live, early apoptosis, late apoptosis, necrosis) based on their positive or negative staining for Annexin V or PI using FlowJo software V10 (Ashland, OR, USA). Annexin V+/PI- cells represent early apoptotic cells, while Annexin V+/PI + cells represent late apoptotic cells. Results are displayed as the percentage of Annexin V-positive (Annexin V+) cells, with the experiment independently repeated three times. The data represent one of three independent experiments.

### Determination of mitochondrial membrane potential (ΔΨm)

The ΔΨm, a measure of electric potential difference (voltage) across the inner mitochondrial membrane, was measured using a non-invasive functional method using a spectrofluorometer (Perkin Elmer LS55) [35, 36]. ΔΨm can be used to determine and to monitor a spontaneous change in mitochondrial function in drug-treated cells [35, 36]. Rhodamine B (Rho-B) was used as a probe to estimate ΔΨm in which the accumulation of Rho-B follows Nernstian distribution across mitochondrial membrane in the cells [37]. In brief, EoL-1 (3.0 × 10^5^ cells/mL) and MV4-11 (1.5 × 10^5^ cells/mL) cells were incubated with four concentrations (IC_10_, IC_20_, IC_30_, IC_50_) of F-EtOAc and F10 for 48 h. Then, cells were harvested, washed with PBS, pH 7.4, and resuspended in HEPES-Na + buffer, pH 7.25, with 40 nM Rho-B in 1 cm quartz cuvette vigorously stirred at 37 °C. The Rho-B fluorescence (F) at 582 nm (excited at 553 nm) was monitored as a function of time. Then 200 µM MTT was added to the cells, yielding a progressive decrease in Rho-B fluorescence which was due to the translocation of Rho-B from outside to inside mitochondria. Indeed, the reduction of MTT to produce formazan, a Rho-B quencher, was located exclusively in the mitochondrial matrix, therefore only Rho-B located in this compartment was quenched [37]. The accumulation of Rho-B in the matrix, is augmented in a ΔΨm-driven manner, as predicted by the following Nernst equation:

ΔΨm =– 61.51 logVi– 258.46 mV.

where, Vi is largely empirical, representing the mitochondrial dye Rho-B concentration: Vi = ($\:\frac{\varDelta\:\varvec{F}}{\varDelta\:\varvec{t}}$) x ($\:\frac{\varvec{C}\varvec{T}}{\varvec{F}0}$) nM/sec. During a very small ∆t after the addition of MTT, the rate of change of Rho-B fluorescence intensity (∆F) with respect to time (∆t) was determined as the slope of the tangent to the fluorescence curve F = f(t). CT was a Rho-B concentration factor, F0 was the initial Rho-B fluorescence intensity at t = 0, and– 258.46 mV was a constant unit in millivolts (mV). In the present study, ΔΨm values were presented as absolute|ΔΨm| values [37].

### Western blotting analysis

EoL-1 and MV4-11 cells were used as cell models to evaluate the expression level of proteins after incubated with three non-cytotoxic (IC_10_, IC_15_, IC_20_) concentrations of F-EtOAc and F10 for 48 h. Following lysis of the cells using radioimmunoprecipitation (RIPA) buffer (50 mM Tris-HCl, 150 mM NaCl, 1% Triton X-100, 1.0 mM EDTA, 0.1% SDS) in the presence of protease and phosphatase inhibitors, the protein concentration was determined by the Pierce™ BCA Protein Assay Kit (ThermoFisher Scientific, IL, USA). Extracted protein samples at 30–60 µg were separated by 7.5% and 12% sodium dodecyl-sulfate polyacrylamide gel electrophoresis (SDS-PAGE) method based on molecular size of the protein, then transferred onto polyvinylidene difluoride (PVDF) membranes. FLT3, WT1, p53, cleaved caspase-3 and GAPDH protein detection (Table S1) were performed as previously described [38]. The specific protein bands were determined by Luminata™ Forte Western HRP substrate and exposed to X-ray film (Sakura, Japan). The Quantity One 1-D Analysis software (Bio-Rad, USA) was used to quantify band density. The density values of target protein bands were normalized to GAPDH bands.

### Statistical analysis

All data were expressed as the mean ± standard deviation (SD) from three independent experiments. The levels of cell populations in the treatment groups were compared with the vehicle control in each experiment. Statistical analysis was performed using SPSS software Ver. 22 (SPSS Inc., USA). The statistical differences between means were analyzed using one-way ANOVA. Differences were considered significant when the p-value was less than 0.05 (*p < 0.05).

## Results

### Yield of *G. pentaphyllum* crude fractional extracts

**Fig. 1:**
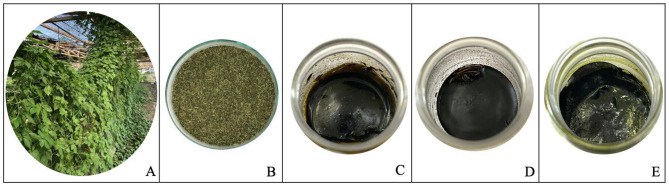
(**A**) Image of the plant *Gynostemma pentaphyllum* in its natural form, (**B**) powdered dried leaves prepared for extraction, (**C**) hexane fractional extract (F-Hex), (**D**) ethyl acetate fractional extract (F-EtOAc), and (**E**) ethanol fractional extract (F-EtOH) obtained from the maceration process

The maceration of *G. pentaphyllum* leaf samples resulted in three groups of crude fractional extracts, including non-polar (F-Hex), intermediate polar (F-EtOAc), and polar (F-EtOH) extracts (Fig. 1). The physical characteristics of each extract were pasty greenish semi-solid masses at room temperature. The F-EtOH extract showed the highest percent yield (%w/w) at 2.70 ± 0.06%, followed by F-EtOAc at 1.60 ± 0.35% and F-Hex at 0.58 ± 0.04%. These differences in the yield indicate specific affinities between the solvents and the chemical compounds in the plant, affecting their extraction efficiencies, as reported by Chen et al. (2016) [39].

### Cytotoxicity of crude fractional extracts on leukemia cell models

**Fig. 2:**
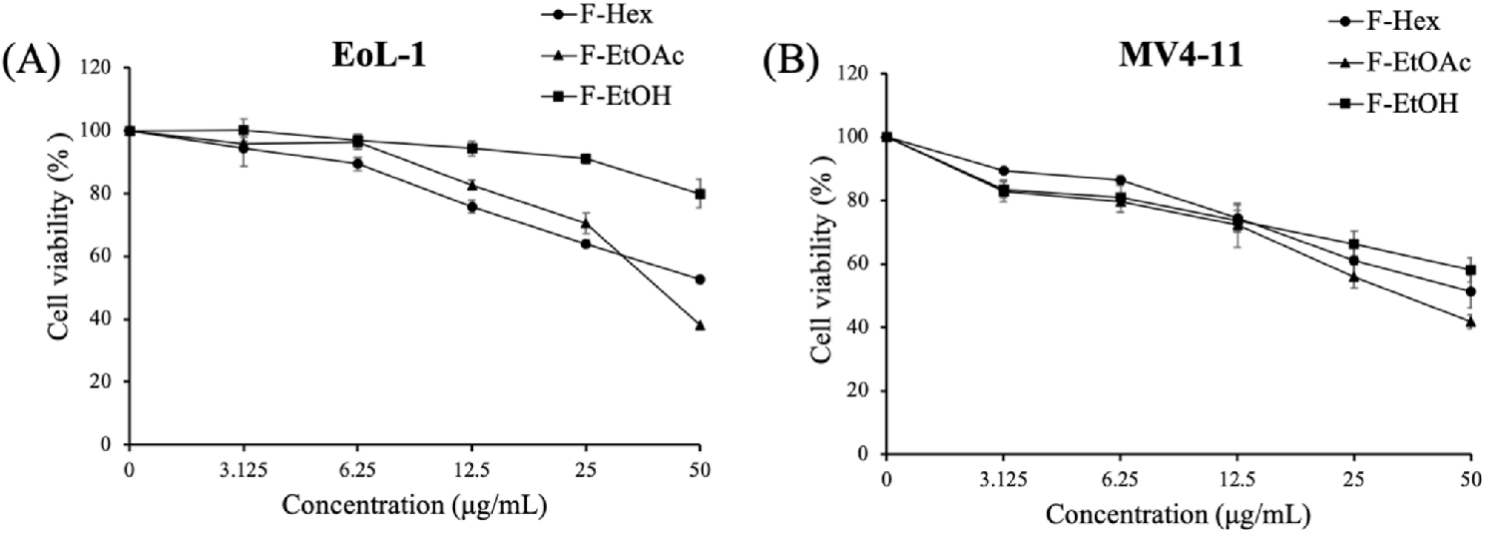
Cytotoxic effects of *G. pentaphyllum* crude fractional extracts on (**A**) EoL-1 and (**B**) MV4-11 leukemic cell lines after 48 h of treatment. Data are presented as mean ± SD from three independent experiments, each performed in triplicate

The in vitro cytotoxicity of crude extracts on various human leukemia cell lines was assessed using the MTT assay. The sensitivity of the cells to each extract was measured as the half-maximal growth inhibitory concentration (IC_50_), which indicates the effectiveness of the extract in inhibiting a specific biological process in the cells [40]. The results demonstrated variations in cytotoxicity among the extracts and cell lines. Leukemia cell lines vary in their origin, morphology, and genomes, leading to differences in their susceptibility to therapeutic agents. F-EtOAc exhibited the highest activity with IC_50_ values of 40.82 ± 0.8 µg/mL in EoL-1 cells and 35.54 ± 4.1 µg/mL in MV4-11 cells (Fig. 2) when compared to the other extracts with IC_50_ values of > 50 µg/mL. According to the National Cancer Institute (NCI) guidelines, crude extract was identified as highly cytotoxic if IC_50_ < 10 µg/mL, moderately cytotoxic if IC_50_ 20 ≤ 50 µg/mL, and weakly cytotoxic if IC_50_ > 50 µg/mL [41].

### Isolation and purification of F-EtOAc extract and identification of isolated compounds

**Fig. 3:**
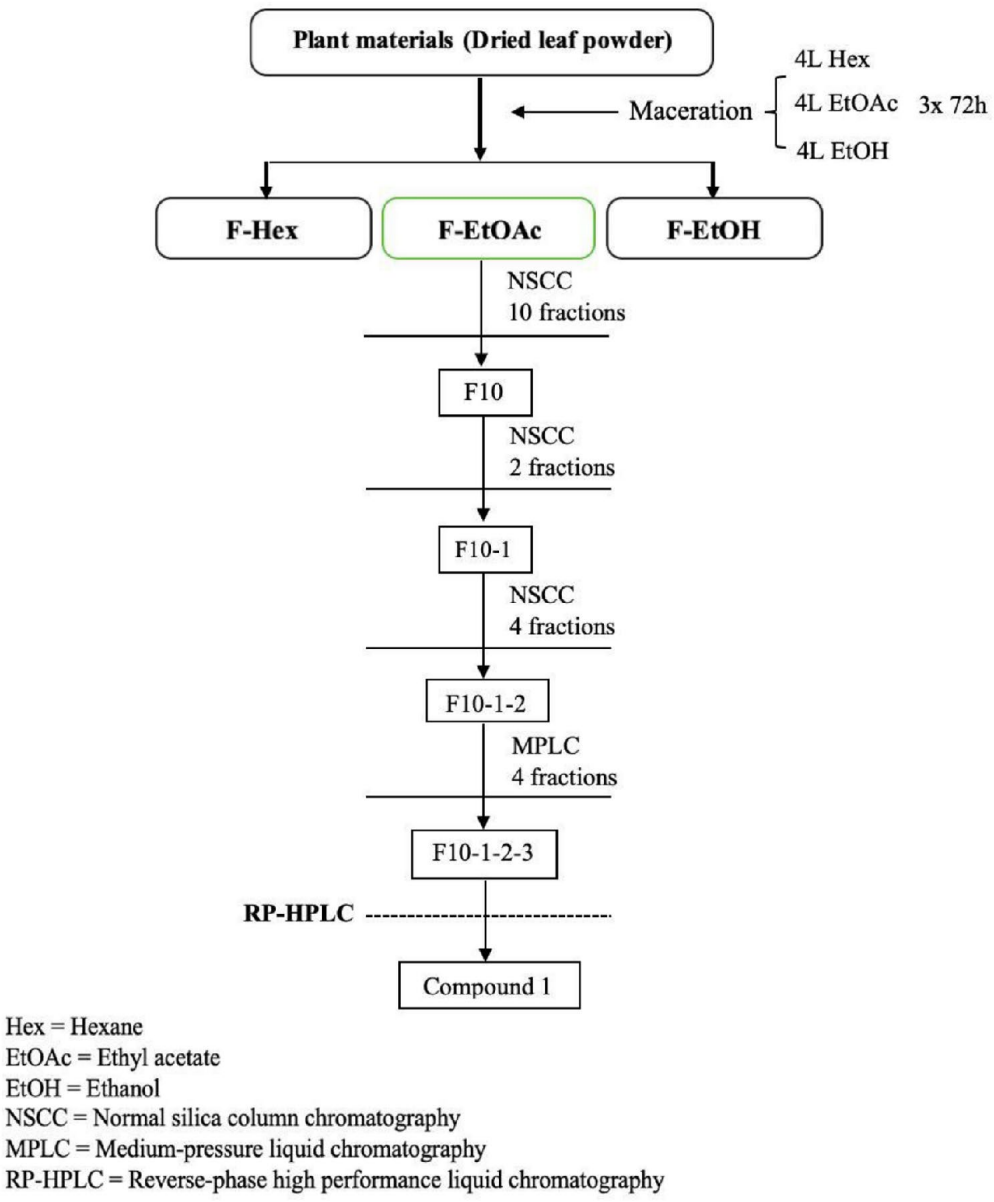
Scheme illustrating entire isolation and purification process of F-EtOAc extract

As shown in Fig. 3, F-EtOAc extract (4.8 g) was separated using NSCC with a hexane/ethyl acetate solvent mixture (ratios from 100:0 to 0:100), resulting in ten main fractions confirmed by TLC. The yields of each fraction are detailed in Table S2. Fraction 10 (F10) was further subjected to NSCC with the same solvent system. Two major fractions were collected and fraction F10-1 was subjected to NSCC with the same solvent system, yielding four major fractions. Fraction F10-1-2 was further separated using MPLC. Four major fractions were collected from this process, and subsequent purification of F10-1-2-3 was performed using RP-HPLC system. The target compound 1, present in F10, was obtained from F10-1-2-3 as a light-yellow shapeless powder and submitted for spectroscopic analyses. ESI-MS calcd for C_13_H_18_O_3_Na, with a molecular weight of 222.2802 g/mol, found *m*/*z* 245.1158 [M + Na]^+^ (Fig. S1). ^1^H-NMR spectrum (Fig. S2) with a literature search identified compound **1** as dehydrovomifoliol [42, 43].

### Cytotoxicity of F-EtOAc isolated fractions by MTT assay

This study aimed to identify the most potent extract, hypothesizing that significant activity in the active crude extract might become more prominent when purified. F-EtOAc was further fractionated, with fraction F10 showing the strongest activity with IC_50_ values of 9.68 ± 0.6 µg/mL in EoL-1 cells and 16.33 ± 1.3 µg/mL in MV4-11 cells, while other fractions possessed minimum to moderate activity (Table 1).

**Table 1:** Cytotoxicity of F-EtOAc fractions on EoL-1 and MV4-11 leukemic cell lines. Data are presented as mean ± sd from three independent experiments, each performed in triplicate

F-EtOAc fractions and drug	IC_50_ value (µg/mL)	
	EoL-1	MV4-11
F1	> 50	> 50
F2	> 50	> 50
F3	> 50	> 50
F4	> 50	> 50
F5	> 50	> 50
F6	> 50	> 50
F7	40.31 ± 1.1	> 50
F8	36.35 ± 1.7	> 50
F9	12.80 ± 2.1	25.87 ± 0.5
F10	9.68 ± 0.6	16.33 ± 1.3
Doxorubicin	0.09 ± 0.5	0.12 ± 0.4

PBMCs are crucial components of the human immune system and are frequently used in toxicology studies as a model to assess cytotoxicity in normal cells. Many studies use PBMCs to investigate the effect of chemicals or extracts on normal cell proliferation [44, 45]. In this study, the cytotoxic activity of three crude fractional extracts and F10 against PBMCs was evaluated. The data in Fig. S3 show variation in the response of PBMCs to treatment, with F-Hex and F-EtOAc extracts significantly reduce (%) cell viability at 50 µg/mL and 100 µg/mL concentrations compared to 0 µg/mL (*p* < 0.001). However, the IC_50_ values remain above 100 µg/mL, confirming that these extracts do not exhibit cytotoxicity in normal PBMCs.

### Effects of F-EtOAc and F10 on cell cycle progression

**Fig. 4:**
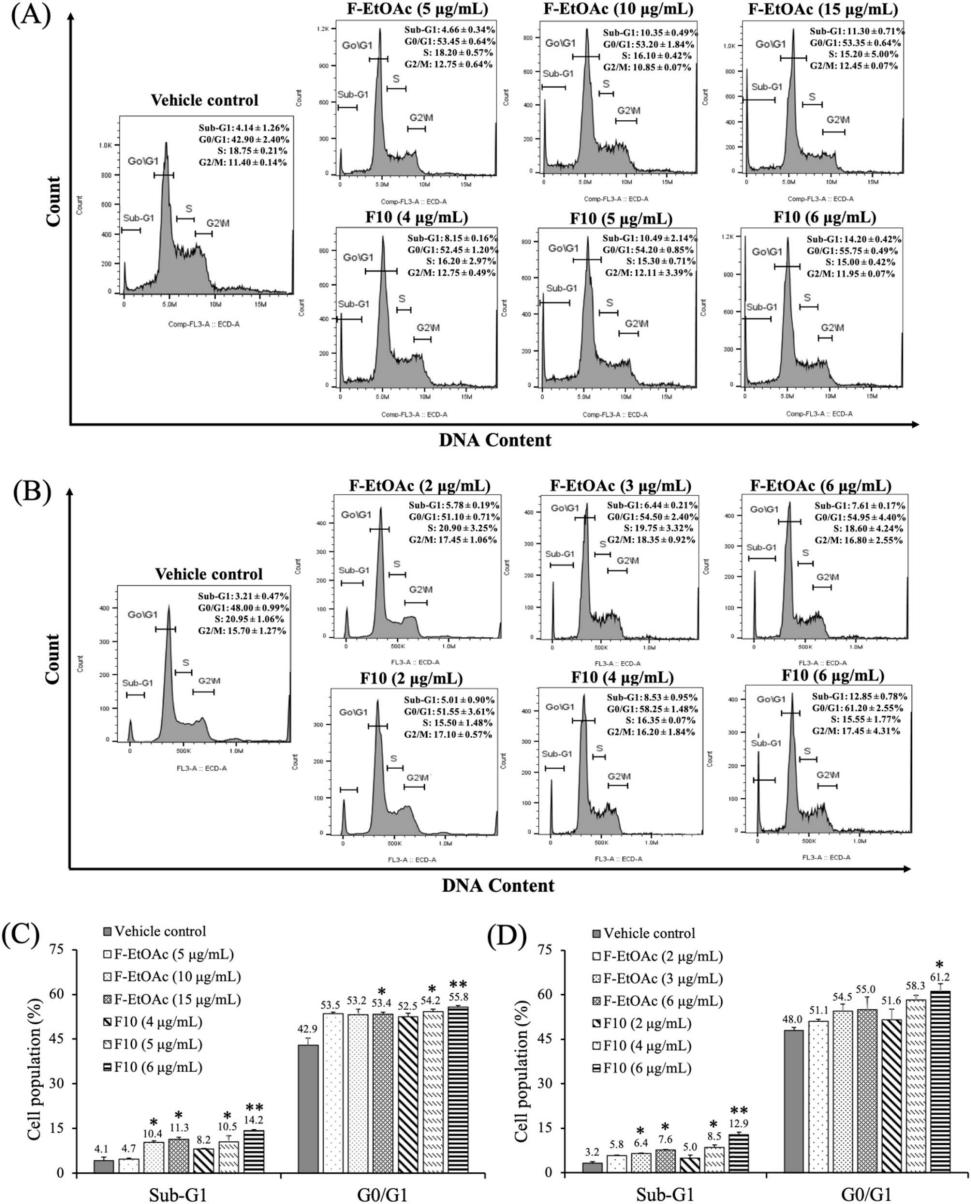
Cell cycle distribution of (**A**) EoL-1 and (**B**) MV4-11 cells after incubation with IC_10_, IC_15_, and IC_20_ concentrations of F-EtOAc and F10 for 48 h. Cell distribution along cell-cycle phases was investigated by flow cytometry analysis of the DNA content after PI staining. Each bar graph shows the cell cycle phases of (**C**) EoL-1 and (**D**) MV4-11 cells after incubation with F-EtOAc and F10 for 48 h in comparison with vehicle control (VC). The bars represent the mean ± SD of three independent experiments. The asterisk (*) indicates significant difference compared to VC; (*p < 0.05, **p < 0.001, one-way ANOVA)

To investigate the molecular mechanism(s) involved in the observed growth inhibition, the cell cycles of EoL-1 and MV4-11 cells exposed to F-EtOAc and F10 at three non-cytotoxic concentrations (IC_10_, IC_15_, IC_20_) were examined through flow cytometric analysis. As shown in Fig. 4, apparent dose-dependent increase in sub-G1 peaks were found in both cell lines following treatment, indicating apoptotic cells with hypodiploid DNA content [46]. Cells incubated with IC_20_ dose of F10 (6 µg/mL) for 48 h showed a significantly higher percentage of apoptotic cells in the sub-G1 phase compared to the vehicle control group in both EoL-1 and MV4-11 cells (*p* < 0.001). A dose-dependent increased in cells at the G0/G1 phase was also observed, particularly significantly increased at the IC_20_ dose of F10 (6 µg/mL) after 48 h of treatment, compared to the control group in EoL-1 (*p* < 0.001) and MV4-11 (*p* < 0.05) cells. The results suggest that F-EtOAc and F10 possess anti-proliferative activity against FLT3-overexpressing AML cells, with the apoptosis pathway being involved.

### Effects of F-EtOAc and F10 on FLT3 and WT1 protein expression and cell proliferation

**Fig. 5:**
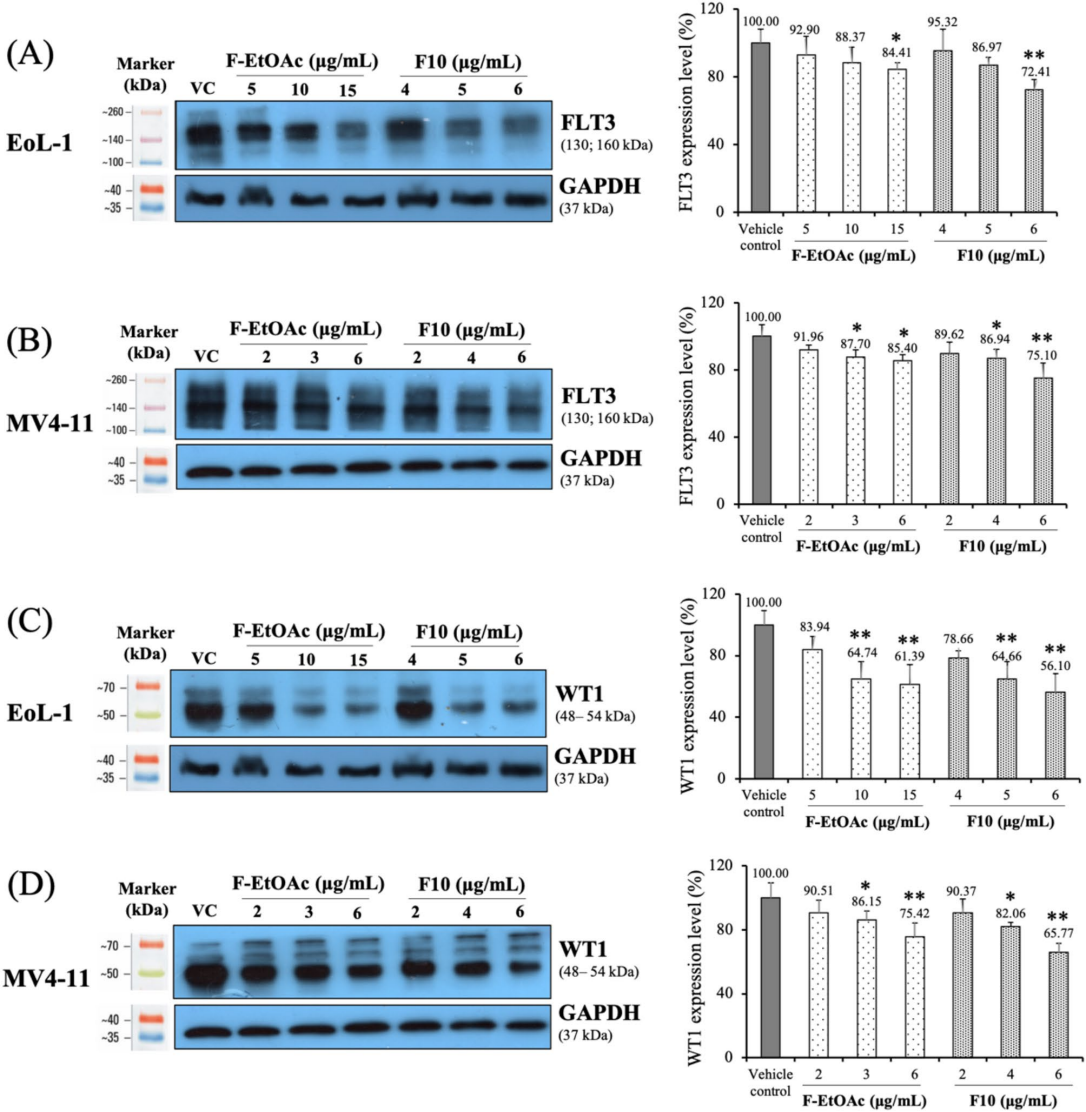
Expression levels of (**A** and **B**) FLT3 and (C and D) WT1 protein after 48 h of exposure to DMSO (vehicle control; VC), IC_10_, IC_15_, and IC_20_ concentrations of F-EtOAc and F10 in EoL-1 and MV4-11 cells. GAPDH was used as a loading control. Histograms represent the mean ± SD of three independent experiments. The asterisk (*) indicates significant difference compared to VC; (*p < 0.05, **p < 0.001, one-way ANOVA)

In this study, FLT3 and WT1 protein expression was used as markers of leukemia cell proliferation and was determined using Western blotting. EoL-1 and MV4-11 cell lines, which exhibit high levels of FLT3 and WT1 protein expression, were used as the cell models [32, 47]. The results showed that both F-EtOAc and F10 disrupted FLT3 and WT1 activities in a dose-dependent manner, influencing cell proliferation in both cell lines, as shown in Fig. 5. A significant reduction in FLT3 (Fig. 5A and B) and WT1 (Fig. 5C and D) expression was noticeable at the IC_20_ concentration of F-EtOAc and F10 in both EoL-1 and MV4-11 cells (*p* < 0.001), demonstrating substantial inhibitory effects compared to the vehicle control after 48 h of treatment. These results correlated with the effects on total viable cell numbers, which were confirmed using the trypan blue exclusion test, as shown in Fig. S4.

### Effects of F-EtOAc and F10 on cell apoptosis

**Fig. 6:**
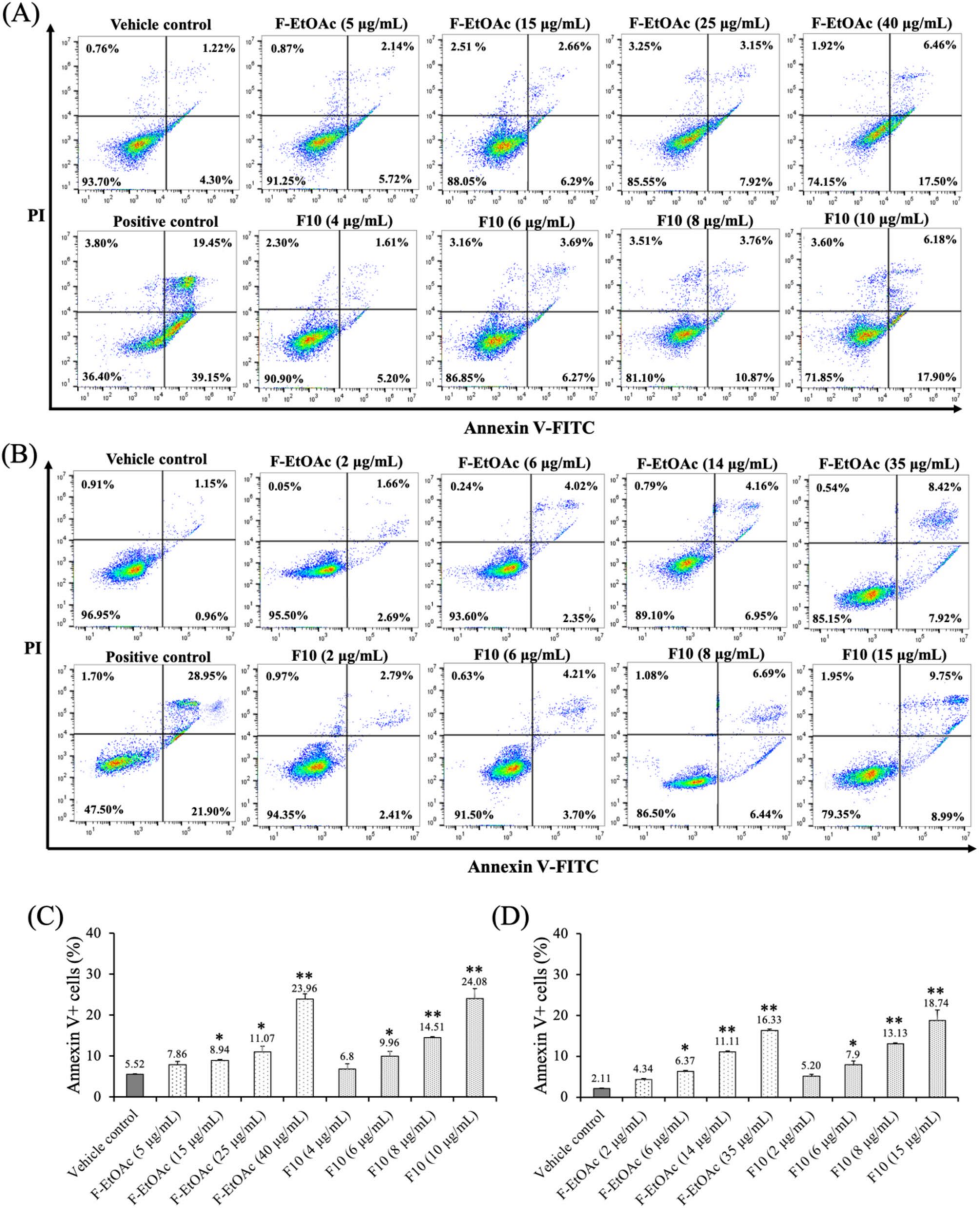
Apoptosis induction activity after 48 h of exposure to IC_10_, IC_20_, IC_30_ and IC_50_ concentrations of F-EtOAc and F10 on (**A**) EoL-1 and (**B**) MV4-11 cells. The percentages represent the proportion of total events within each respective categories, as gated in the flow cytometry plots. The bar graphs represent Annexin V + cells (%) which are in early and late apoptosis as determined by Annexin V/PI staining, analyzed by flow cytometry after incubation with F-EtOAc and F10 on (**C**) EoL-1 and (**D**) MV4-11 cells. Doxorubicin was used as positive control (PC) in this experiment. The percentage of apoptotic cells in each treatment was statistically compared with vehicle control (VC). The bars represent the mean ± SD of three independent experiments. The asterisk (*) indicates significant difference compared to VC; (*p < 0.05, **p < 0.001, one-way ANOVA)

The Annexin V/PI double staining assay was used to confirm the apoptotic induction activity of F-EtOAc and F10 on EoL-1 and MV4-11 cells, as indicated by the sub-G1 peaks in the cell cycle distribution. The percentage of Annexin V + apoptotic cells was measured after incubating EoL-1 and MV4-11 cells with IC_10_, IC_20_, IC_30_ and IC_50_ concentrations of F-EtOAc and F10 for 48 h. Doxorubicin at its IC_50_ concentration was used as a positive control (Table 1). A substantial increased in the percentage of Annexin V + cells was observed after incubated with both F-EtOAc and F10, as summarized in Fig. 6. Both early (Annexin V+/PI- quadrant) and late (Annexin V+/PI + quadrant) stages of EoL-1 and MV4-11 cells increased in a dose-dependent manner. Specifically, the proportion of Annexin V + cells significantly increased after 48 h of treatment with F10 at its IC_50_ concentrations; 10 µg/mL in EoL-1 cells (24.08 ± 2.37%, *p* < 0.001, Fig. 6C) and 15 µg/mL in MV4-11 cells (18.74 ± 2.62%, *p* < 0.001, Fig. 6D), compared to the vehicle control (5.52 ± 0.19% and 2.11 ± 0.13%, respectively). The results suggest that the induction of apoptosis by F-EtOAc and F10 is at least partially responsible for the observed growth inhibition in EoL-1 and MV4-11 leukemic cells.

### Effects of F-EtOAc and F10 on mitochondrial membrane potential (ΔΨm)

**Fig. 7:**
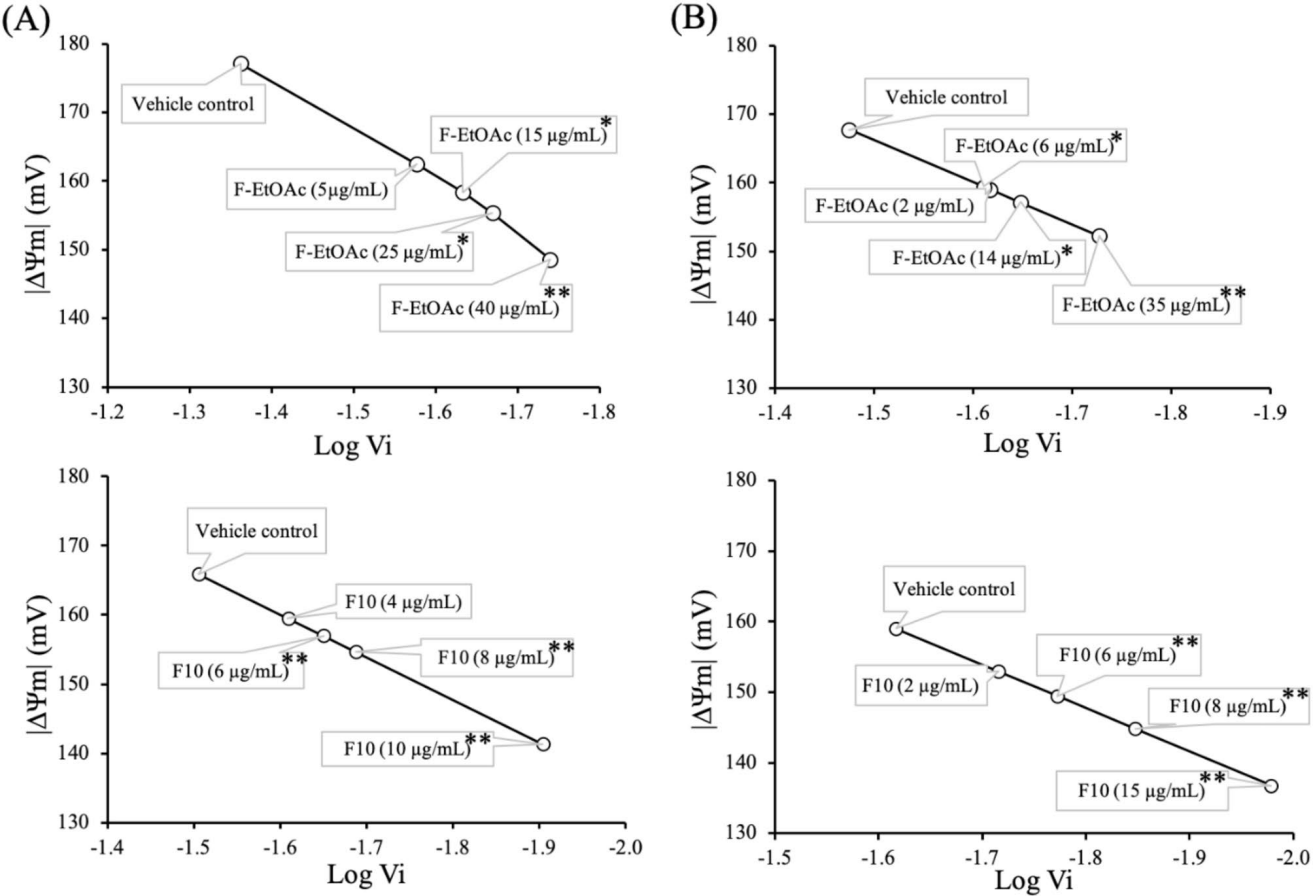
Effect of F-EtOAc and F10 on ∆Ψm after 48 h of exposure to IC_10_, IC_20_, IC_30_ and IC_50_ concentrations in (**A**) EoL-1 and (**B**) MV4-11 cells. Vi represents the mitochondrial Rho-B dye concentration in the inner mitochondrial membrane. A reduction in|ΔΨm| indicates a decrease in the ΔΨm. The data are presented as the mean ± SD of three independent experiments. The asterisk (*) indicates significant difference compared to vehicle control; (*p < 0.05, **p < 0.001, one-way ANOVA)

To determine whether the apoptotic induction activity of F-EtOAc and F10 correlates with changes in mitochondrial function, a spectrofluorometric assay was conducted to evaluate the ΔΨm changes in EoL-1 and MV4-11 cells. The cells were incubated with IC_10_, IC_20_, IC_30_ and IC_50_ concentrations of F-EtOAc and F10 for 48 h, and the results were compared with vehicle control. Figure 7A and B show the linear increase in log Vi associated with a decrease in the absolute value of ΔΨm. Notably, mitochondrial depolarization, indicated by the reduced ΔΨm, was significantly more pronounced in the cells treated with F-EtOAc and F10 at IC_50_ concentrations; 40 µg/mL and 10 µg/mL in EoL-1 cells (Fig. 7A), and 35 µg/mL and 15 µg/mL in MV4-11 cells (Fig. 7B), compared to the vehicle control (*p* < 0.001). These findings suggest that the loss of ΔΨm may contribute to apoptosis induction observed in EoL-1 and MV4-11 cells treated with F-EtOAc and F10.

### Effect of F-EtOAc and F10 on the expression of proteins associated with cell cycle control and apoptosis

**Fig. 8:**
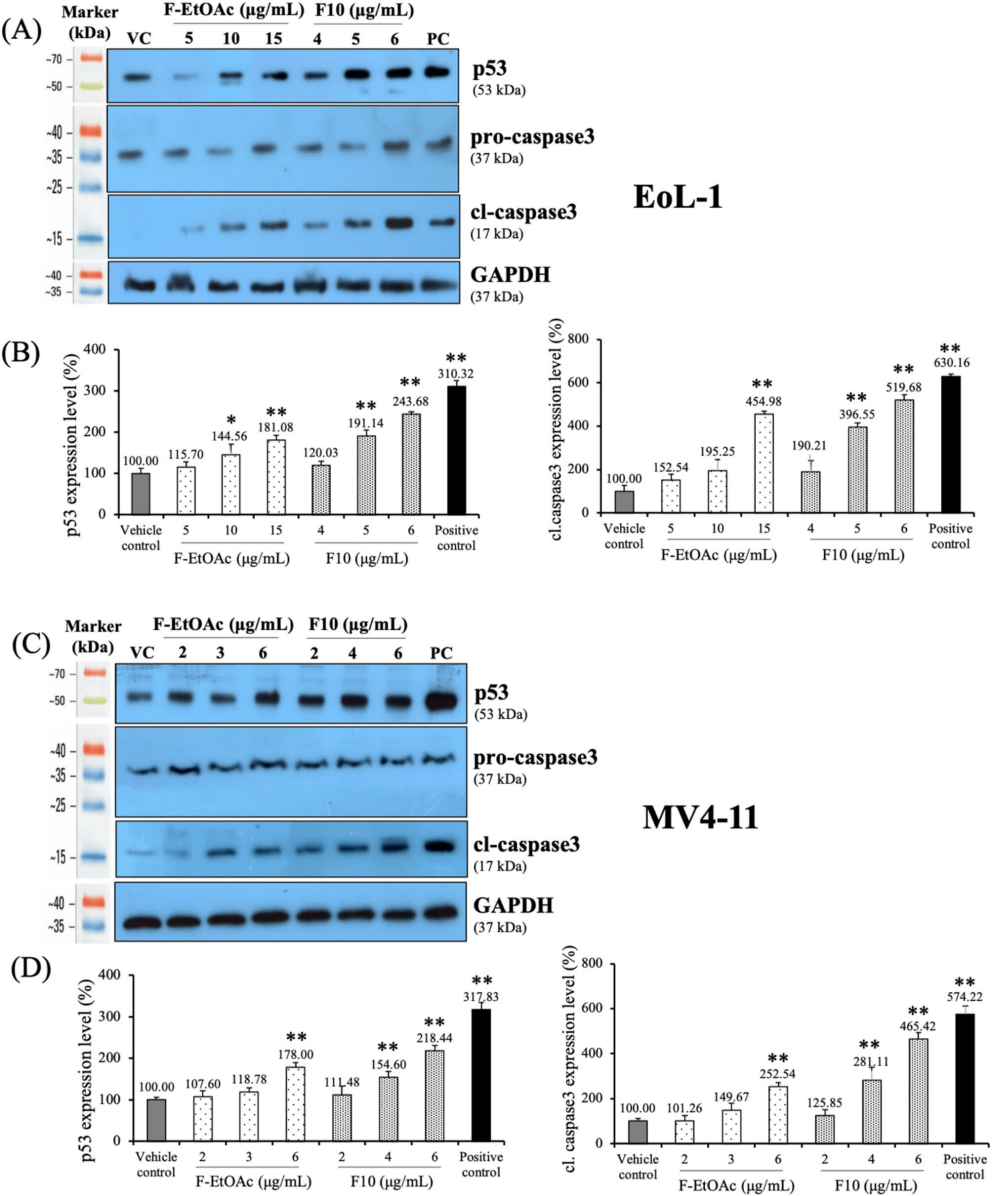
Levels of p53, pro-caspase3 and cleaved-caspase3 protein following 48 h of exposure to IC_10_, IC_15_, and IC_20_ concentrations of F-EtOAc and F10 in (**A** and **B**) EoL-1 cells and (**C** and **D**) MV4-11 cells. GAPDH was used as a loading control. Histograms show the mean ± SD of three independent experiments. Doxorubicin was used as positive control (PC) in this experiment. The asterisk (*) indicates significant difference compared to vehicle control (VC); (*p < 0.05, **p < 0.001, one-way ANOVA)

Based on the observed increase in the number of Annexin V + cells and mitochondrial depolarization, further investigations were conducted after incubating with F-EtOAc and F10 at IC_10_, IC_15_, and IC_20_ concentrations to determine key protein markers associated with cell cycle control and cell apoptosis. Doxorubicin (Dox), an anti-cancer chemotherapeutic agent, disrupts DNA transcription and replication by intercalating between DNA base pairs and stabilizing topoisomerase II, as well as causing oxidative damage [48]. Tumor suppressor p53 activation primarily induces cell-cycle arrest and initiates apoptosis in response to DNA damage, contributing significantly to the cytotoxic effects of anti-cancer agents [48]. Caspase-3 plays an important role in chromatin condensation and DNA fragmentation, the hallmark features of apoptosis [49]. In this experiment, the ability of Dox (at IC_20_ concentration, Table 1) to upregulate the expression of p53 and cleaved caspase-3 proteins was assessed as an established positive control (PC) [46, 50]. The results in Fig. 8 showed that F-EtOAc and F10 influenced the expression of p53 and active caspase-3 protein in both EoL-1 and MV4-11 cells. The levels of p53 and cleaved caspase-3 were significantly increased after incubation with IC_20_ dose of F10 (6 µg/mL) in EoL-1 cells (Fig. 8B) and MV4-11 cells (Fig. 8D), compared to the vehicle control after 48 h of treatment (*p* < 0.001), indicating apoptosis induction.

## Discussion

Most phytochemical studies on *G. pentaphyllum* have focused on the highly polar glycosides extracted with polar solvents such as ethanol, methanol, or water, which contain major anti-cancer components including saponin gypenosides and flavonoids [15, 16, 51]. For instance, Piao et al. [51] extracted *G. pentaphyllum* using ethyl acetate but utilized ethanol and water in subsequent elution and isolation steps, resulting in predominantly polar compounds. In contrast, Li et al. [52] demonstrated that a nonpolar fraction (named EA1.3 A) containing alkaloids, organic esters, terpenes, and catechol substances, extracted with n-hexane and trichloromethane, exhibited potent growth-inhibitory activities against various cancer cell lines, by regulating cell cycle progression and inducing apoptosis. Inspired by these findings, we focused on preparing nonpolar, intermediate polar, and polar fractional extracts of *G. pentaphyllum* leaves through sequential maceration with n-hexane, ethyl acetate, and ethanol, respectively. To further understand the distribution of medicinally relevant compounds in *G. pentaphyllum*, we searched the studies investigating the biosynthesis of bioactive components across different tissues of *G. pentaphyllum*. According to Chen et al. [53] used Unigene analysis to study the gene expression of enzymes involved in triterpenoid saponin biosynthesis essential for medicinal properties of *G. pentaphyllum*. Their results showed that leaves had the highest levels of key enzymes, including FPS (farnesyl pyrophosphate synthase), SS (squalene synthase), and SE (squalene epoxidase), suggesting a higher concentration of triterpenoid saponins, such as gypenosides, in leaves compared to stems and roots. Similarly, Zhao, Ge, and Miao [54] reported distinct metabolite profiles in different tissues of *G. pentaphyllum*, with high flavonoid synthesis in leaves, and increased genes activity associated with the production of sesquiterpenes, triterpenoids, and diterpenoids in stems and leaves. These findings collectively indicate that leaves may undergo more extensive secondary metabolite production, which could contribute to their prominent medicinal effects. Given this evidence, most studies, including ours, have focused on leaves, which appear to be the most promising tissue for anti-cancer research. However, considering that stems may also contain bioactive compounds, investigating the anti-cancer potential of stem tissues would be a valuable direction for future research.

The cytotoxic activity of three crude extracts from *G. pentaphyllum* against a range of human leukemia cell lines; EoL-1, K562, Molt-4, MV4-11, U937, KG1 and KG1-a was evaluated using the tetrazolium-based cell viability assay. Notably, the ethyl acetate (F-EtOAc) extract exhibited the strongest and most selective cytotoxic activity against EoL-1 and MV4-11 cells, both of which overexpress FLT3, with IC_50_ values below 50 µg/mL. This activity was superior compared to the hexane and ethanol extracts (Fig. 2), making F-EtOAc the prime candidate for further fractionation. The observed variations in IC_50_ values may be attributed to the differing sensitivity of various leukemia cell types to the same therapeutic agent. Supporting our findings, Wang et al. [55] reported that the ethyl acetate (EA) fraction of *G. pentaphyllum* exhibited higher anti-cancer activity against A549 cells than the ethanol extract, suggesting enrichment of potent compounds in the EA fraction. Similarly, Ong et al. [56] demonstrated that the ethyl acetate extract of *G. pentaphyllum* had the lowest MIC value (0.08 mg/mL) against Gram-positive *Bacillus cereus* and *Staphylococcus aureus*, indicating its potent antibacterial activity. Among the ten fractions derived from F-EtOAc, fraction F10 exhibited the strongest activity, with IC_50_ values of 9.68 ± 0.6 µg/mL in EoL-1 cells and 16.33 ± 1.3 µg/mL in MV4-11 cells. In contrast, the other fractions displayed minimal to moderate activity (Table 1). Consistent with previous research, almost all cytotoxic compounds and extracts isolated from *G. pentaphyllum* have demonstrated anti-proliferative activities, with IC_50_ values ranging from 0.05 to 74.3 µg/mL [20]. Given that plant crude extracts are complex mixture of bioactive and non-bioactive phytochemicals, their overall potency is often lower than that of isolated fractions or pure chemotherapeutic agents like doxorubicin. This is reflected in the IC_50_ values observed for the chemotherapy drug and fractions in our study (Table 1).

Given the significant cytotoxic activity observed in the F-EtOAc extract and fraction F10 against drug-sensitive EoL-1 and MV4-11 cell lines, we delved deeper into cell cycle progression using flow cytometry to elucidate the molecular mechanism behind the reduced cell viability. Employing three different non-cytotoxic doses, we found that a higher concentration of F10 (6 µg/mL) for 48 h led to an increase in the G0/G1 and sub-G1 phases in both cell lines compared to the control group. This suggests that F10 induces G0/G1 arrest and apoptosis in a dose-dependent manner, with more pronounced effects than F-EtOAc (Fig. 4). Previous studies align with our findings, showing that gypenosides from *G. pentaphyllum* induced G0/G1 arrest in various cancer cell lines, including WEHI-3 mouse leukemia [14], A549 human lung adenocarcinoma [26], HL-60 human myeloid leukemia [27], and Colo 205 human colon cancer [57] cells. Based on the occurrence of sub-G1 population, the Annexin V-FITC/PI assay, ∆Ψm levels, and Western blotting of apoptosis markers (p53 and cleaved caspase-3), we determined that F-EtOAc and F10 might suppress cancer cell proliferation by promoting apoptosis.

Apoptosis, a caspase-dependent programmed cell death, is crucial in cancer therapy for controlling uncontrolled cancer cell growth [58, 59]. It can be triggered either by the activation of death receptors (extrinsic pathway) or by mitochondrial activation (intrinsic pathway) [59]. In this study, F10 treatment significantly increased Annexin V + apoptotic cells at 10 µg/mL in both EoL-1 and MV4-11 cells (Fig. 6C and D) after 48 h, indicating substantial apoptosis. The reduction in ∆Ψm was significantly more pronounced in both cell lines treated with F10 (Fig. 7), suggesting involvement of the intrinsic mitochondrial pathway. Additionally, significant up-regulation of p53 and activation of cleaved caspase-3 were observed with 6 µg/mL of F10 treatment for 48 h in both cell lines (Fig. 8). It has been reported that increased p53 levels are sufficient to activate protein kinase C, which stimulates *p53* gene transcription, causes G1-phase arrest, and promotes cellular repair processes [60]. Our findings suggest that F10 fraction of F-EtOAc extract contains potent anti-cancer compounds that target the proteins associated with cell proliferation and apoptosis pathways, likely contributing to the observed effects on cell cycle phases and apoptosis. Apoptosis is initiated by the activation of cysteine proteases family known as caspases. These caspases are initially produced as inactive pro-enzymes within cells and become biologically active upon cleavage [57]. Our study demonstrated that fraction F10 promoted the activation of caspase-3, a crucial step in apoptosis.

Interestingly, various fractions and active components of *G. pentaphyllum* have also been shown to induce apoptosis. Hsu et al. [14] reported that treating mouse leukemia WEHI-3 cells with gypenosides (150 µg/mL) upregulated p53, increased reactive oxygen species (ROS) and Ca^2+^ production, and decreased ∆Ψm levels. This cascade led to cytochrome c, AIF, and Endo G release, activating initiator caspases-8 and − 9, and subsequently cleaving effector caspase-3, triggering apoptosis. Similarly, Lin et al. [27] found that treating HL-60 myeloid leukemia cells with gypenosides (150 µg/mL) resulted in G0/G1 arrest. This was accompanied by the down-regulation of p21, p16, p27, cyclin D1 and cyclin E, as well as the inhibition of Cdk2 and Cdk6. The gypenosides-induced sub-G1 apoptosis was linked to increased ROS and Ca^2+^ levels, decreased ∆Ψm, release of cytochrome c, activation of caspase-9 and − 3, alongside the release of AIF and Endo G [27]. Moreover, other active components of *G. pentaphyllum*, such as flavonoids [24] and water extract [61], have been reported to induce apoptosis in tumor cells by regulating the Bcl-2 protein family.

FLT3 is recognized as a pivotal protein marker in AML patients, with its overexpression leading to ligand-independent activation and phosphorylation of the receptor. This results in constitutive kinase activity, providing proliferative and survival advantages to AML cells [62]. Given its critical role in AML progression, FLT3 is a major target for cancer therapy. Ly et al. [63] demonstrated that green tea polyphenols suppress the proliferation of FLT3-mutated AML cells through suppression of FLT3 expression, thereby inducing apoptotic cell death. In our study, EoL-1 cells, which express wild-type FLT3, require FLT3 ligand for activation, while MV4-11 cells harbor the FLT3/ITD mutation, resulting in a constitutively active FLT3 kinase [6, 32, 62]. WT1 protein is expressed in the majority of AML cases, and its overexpression has been reported to play a significant prognostic role in this disease [7, 8]. Tima et al. [34] reported that extracts from *Cordyceps militaris* showed inhibitory effects on WT1 protein expression and cell proliferation in EoL-1 leukemic cells. Building on these insights, our study demonstrated that active compounds in fraction F10 from *G. pentaphyllum* may inhibit leukemic cell proliferation by suppressing FLT3 and WT1 expression. Western blotting analysis revealed that treatment with F10 (6 µg/mL) for 48 h significantly reduced FLT3 and WT1 expression levels in both EoL-1 and MV4-11 cells (Fig. 5). This suppression was corroborated by a lower viable cell count using trypan blue exclusion (Fig. S4), indicating decreased cell proliferation. These findings suggest that F10 reduce the viability of AML cells through mechanisms affecting FLT3 and WT1 function. However, further detailed studies are required to confirm this hypothesis. To our knowledge, there have been no reports to date on the effects of *G. pentaphyllum* on FLT3 and WT1 protein expression in leukemic cells.

Given the significant activities observed in F-EtOAc extract and fraction F10, we subjected F10 to bioassay-directed fractionation. This process led to the isolation of compound 1 (Fig. 3) using multiple chromatographic columns. Bioassay-guided fractionation is a method that separates and analyze compounds of interest from a complex extract mixture, with sequential testing for biological activity [55]. During the initial cytotoxicity screening, sub-fractions F10-1, F10-1-2 and F10-1-2-3 (Fig. 3) were identified for further purification based on their efficacy against EoL-1 and MV4-11 cells, indicated by their IC_50_ < 10 µg/mL (Table S3) [41]. The structure of the purified compound 1, obtained from F10, was elucidated and confirmed by comparing its ^1^H and/or ^13^C NMR data with previously published data, matching the structure of dehydrovomifoliol. This compound, classified as sesquiterpene or megastigmane glycoside, was first identified in 1977 from *Oryza sativa* [42, 43]. Dehydrovomifoliol has also been isolated from the ethyl acetate-soluble part of the methanol extract of *Cucumis sativus* L. leaves, a member of the Cucurbitaceae family [43], and from the leaves of *Nitraria sibirica* Pall., an edible fruit-bearing shrub widely distributed in the Middle East, Central Asia, and northwest China [64]. Moreover, dehydrovomifoliol, when purified from *Solanum lyratum* Thunb. (Solanaceae), demonstrated significant cytotoxic activities against various human cancer cell lines, including HONE-1 nasopharyngeal, KB oral epidermoid carcinoma, and HT29 colorectal carcinoma cells, with IC_50_ values being in the range 4.0–5.7 µM [65]. Recent studies have also highlighted the potential of dehydrovomifoliol from *Artemisia frigida* Willd, a traditional Chinese medicine (TCM), in preventing non-alcoholic fatty liver disease (NAFLD) by influencing hepatic lipogenesis and fatty acid oxidation, identifying *E2F1* as a core gene in NAFLD treatment and linking its role to the regulation of the AKT/mTOR signaling pathway [66]. Additionally, dehydrovomifoliol from *Artemisia frigida* has shown promise in affecting hepatic lipogenesis and fatty acid oxidation via the PPARα–FGF21 signaling axis [67]. To support our findings in this study, we applied an in silico approach using network pharmacology to predict and explore the molecular interactions and pathways through which dehydrovomifoliol may exert its anti-leukemic activity. This analysis identified several key gene targets that dehydrovomifoliol might influence in the context of leukemia, including *TNF*,* AKT1*,* IL6*,* ALB*,* HSP90AA1*,* EGFR*,* ESR1*,* MAPK3*,* CASP3*, and *SRC* (Fig. S5). Furthermore, the Gene Ontology (GO) and Kyoto Encyclopedia of Genes and Genomes (KEGG) pathway analyses indicate that the potential anti-leukemic activity of dehydrovomifoliol may involve pathways associated with signal transduction, cell proliferation, cellular apoptosis, and cell cycle progression (Fig. S5). These pathways are crucial in leukemia pathogenesis and are consistent with the biological processes affected by the components in the F-EtOAc fraction. This supports our findings and provides a foundation for future experimental investigation into therapeutic potential of dehydrovomifoliol. Further studies are necessary to explore the role and underlying mechanisms of the ethyl acetate extract of *G. pentaphyllum* in AML cells, to validate and deepen our understanding of its observed anti-leukemic activities in this study.

## Conclusion

**Fig. 9:**
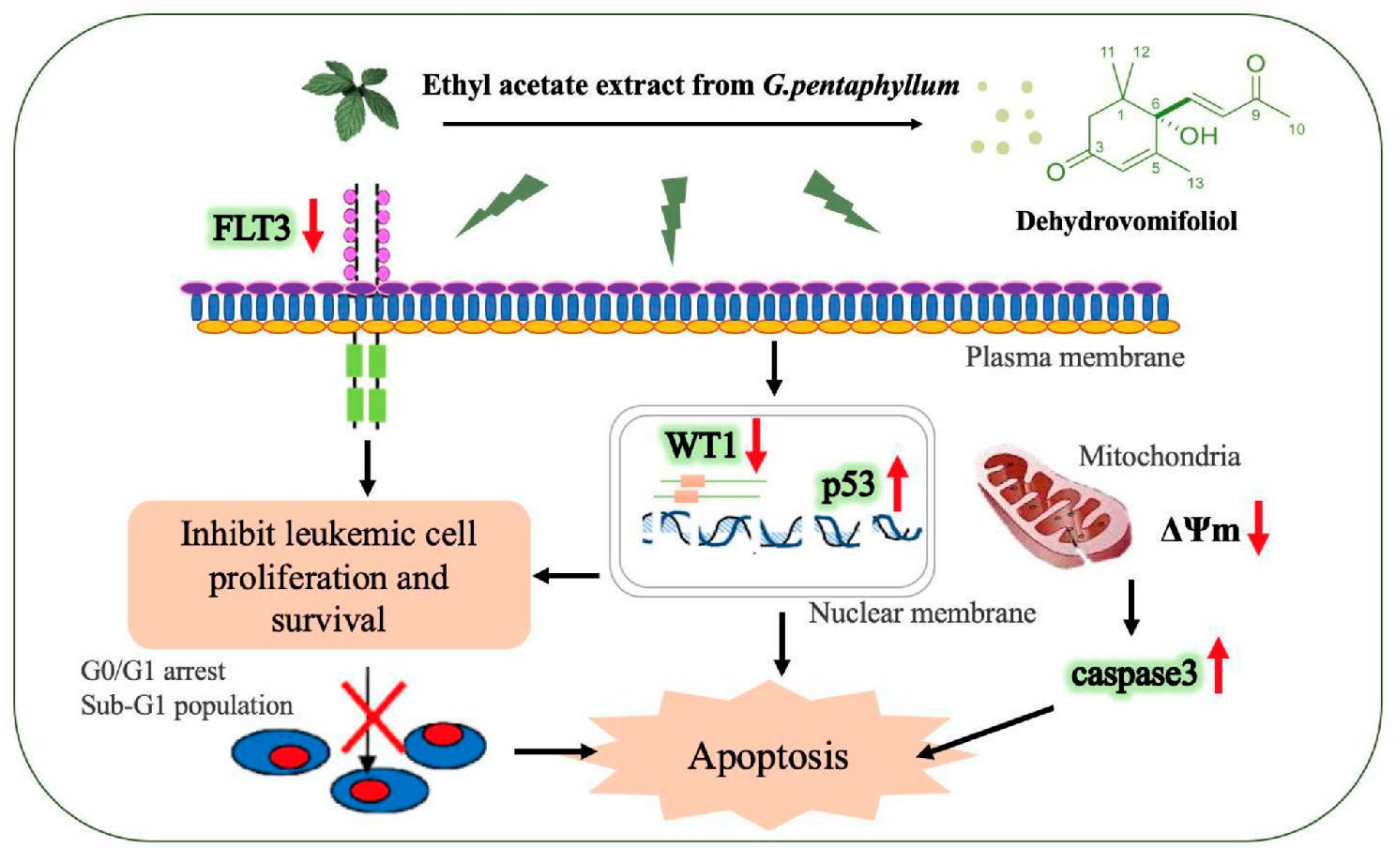
Proposed signaling pathways of the anti–leukemia activity of *Gynostemma pentaphyllum* ethyl acetate extract against EoL-1 and MV4-11 cells

This study demonstrates that the ethyl acetate (F-EtOAc) extract and its active fraction F10 of *Gynostemma pentaphyllum* exhibit significant anti-leukemia activities. The extract and fraction inhibit FLT3 and WT1 expression, induce G0/G1 cell cycle arrest, and trigger mitochondrial depolarization (a decrease in ∆Ψm). Additionally, up-regulate p53 and activate caspase-3, leading to apoptosis. ESI-MS and ^1^H-NMR analyses identified dehydrovomifoliol as the major bioactive compound present in F-EtOAc and F10. A proposed mechanism of action for these effects is illustrated in Fig. 9. Taken together, these findings provide new insights into the mechanisms of action of *G. pentaphyllum*, and highlight its potential against acute myeloid leukemia.

## Electronic supplementary material

Below is the link to the electronic supplementary material.

Supplementary Material 1

Supplementary Material 2

## Data Availability

The datasets supporting the conclusions of this article are included within the article (and its supplementary and additional file(s)). The data generated and/or analyzed during this study are available from the corresponding author upon reasonable request.
