# Supplementary material for: Anti-leukemia activity of the ethyl acetate extract from Gynostemma pentaphyllum (Thunb.) leaf against FLT3-overexpressing AML cells and its phytochemical characterization
Source: BMC Complement Med Ther. 2025 May 13;25:172. doi: 10.1186/s12906-025-04903-0 (PMC12076849; doi:10.1186/s12906-025-04903-0)
Supplement: Supplementary file 1 — Supplementary Material 1 [file 12906_2025_4903_MOESM1_ESM.docx]

**Anti-leukemia activity of the ethyl acetate extract from *Gynostemma pentaphyllum* (Thunb.) leaf against FLT3-overexpressing AML cells and its phytochemical characterization**

Khin Khin Gyi^1,2,3^, Songyot Anuchapreeda^1,4,5^, Nutjeera Intasai^1,4^, Montree Tungjai^6^, Siriporn Okonogi^5,7^, Arihiro Iwasaki^8^, Toyonobu Usuki^3*^, and Singkome Tima^1,4,5*^

*^1^Department of Medical Technology, Faculty of Associated Medical Sciences, Chiang Mai University, Chiang Mai 50200, Thailand*

*^2^Ph.D. Degree Program in Biomedical Sciences, Faculty of Associated Medical Sciences, Chiang Mai University, Under The CMU Presidential Scholarship, Chiang Mai 50200, Thailand*

*^3^Department of Materials and Life Sciences, Faculty of Science and Technology, Sophia University, 7-1 Kioicho, Chiyoda-ku, Tokyo 102-8554, Japan*

*^4^Cancer Research Unit of Associated Medical Sciences (AMS CRU), Faculty of Associated Medical Sciences, Chiang Mai University, Chiang Mai 50200, Thailand*

*^5^Center of Excellence in Pharmaceutical Nanotechnology, Chiang Mai University, Chiang Mai 50200, Thailand*

*^6^Department of Radiologic Technology, Faculty of Associated Medical Sciences, Chiang Mai University, Chiang Mai 50200, Thailand*

*^7^Department of Pharmaceutical Sciences, Faculty of Pharmacy, Chiang Mai University, Chiang Mai 50200, Thailand*

*^8^Department of Applied Chemistry, Faculty of Science and Engineering, Chuo University, 1-13-27 Kasuga, Bunkyo-ku, Tokyo 112-8551, Japan*

**Correspondence: singkome.tima@cmu.ac.th and t-usuki@sophia.ac.jp*

**Table S1. Dilution and reference of the antibodies used for Western Blot (WB).**

| **Target** | **Supplier** | **Reference** | **Dilution** |
| --- | --- | --- | --- |
| FLT3 | Abcam | ab245116 | 1:1000 (WB) |
| WT1 | Cell signaling Technology | 83535 | 1:1000 (WB) |
| GAPDH | Abcam | ab9485 | 1:16,000 (WB) |
| p53 | Affinity Biosciences | AF0879 | 1:1000 (WB) |
| Caspase-3 | Affinity Biosciences | AF6311 | 1:1000 (WB) |
| Cleaved caspase-3 | Cell signaling Technology | 9661 | 1:1000 (WB) |
| Anti-rabbit IgG HRP-conjugated | Abcam | ab205718 |  |

**Table S2. Percentage yield of F-EtOAc isolated fractions from column chromatography.**

| **F-EtOAc Sample** | **Yield (%)** |
| --- | --- |
| F1 | 4.5 |
| F2 | 3.7 |
| F3 | 9.4 |
| F4 | 6.9 |
| F5 | 2.5 |
| F6 | 1.8 |
| F7 | 1.3 |
| F8 | 1.7 |
| F9 | 3.8 |
| F10 | 2.4 |

**Table S3. Cytotoxicity of F10 fractions on EoL-1 and MV4-11 leukemic cell lines performed by MTT assay.** Data are presented as mean ± SD from three independent experiments, each performed in triplicate.

| **F10 fractions** | **IC_50_ value (μg/mL)** | |
| --- | --- | --- |
|  | **EoL-1** | **MV4-11** |
| F10-1 | 7.73 ± 0.1 | 7.04 ± 0.8 |
| F10-2 | 36.41 ± 1.0 | 20.25 ± 0.2 |
| F10-1-1 | 10.77 ± 0.3 | 10.71 ± 0.1 |
| F10-1-2 | 5.78 ± 0.5 | 6.15 ± 0.1 |
| F10-1-3 | >50 | >50 |
| F10-1-4 | 16.72 ± 1.4 | 30.81 ± 2.3 |
| F10-1-2-1 | >50 | >50 |
| F10-1-2-2 | >50 | >50 |
| F10-1-2-3 | 5.63 ± 0.7 | 5.96 ± 0.1 |
| F10-1-2-4 | 39.49 ± 0.5 | 32.10 ± 0.2 |

**Table S4. The inhibitory concentrations of F-EtOAc and F10 using for treatment of EoL-1 and MV4-11 leukemic cell lines.**

| **Treatment** | **Inhibitory concentration (IC) values (μg/mL)** | |
| --- | --- | --- |
|  | **EoL-1** | **MV4-11** |
| F-EtOAc at IC_10_ | 5 | 2 |
| F-EtOAc at IC_15_ | 10 | 3 |
| F-EtOAc at IC_20_ | 15 | 6 |
| F-EtOAc at IC_30_ | 25 | 14 |
| F-EtOAc at IC_50_ | 40 | 35 |
| F10 at IC_10_ | 4 | 2 |
| F10 at IC_15_ | 5 | 4 |
| F10 at IC_20_ | 6 | 6 |
| F10 at IC_30_ | 8 | 8 |
| F10 at IC_50_ | 10 | 15 |


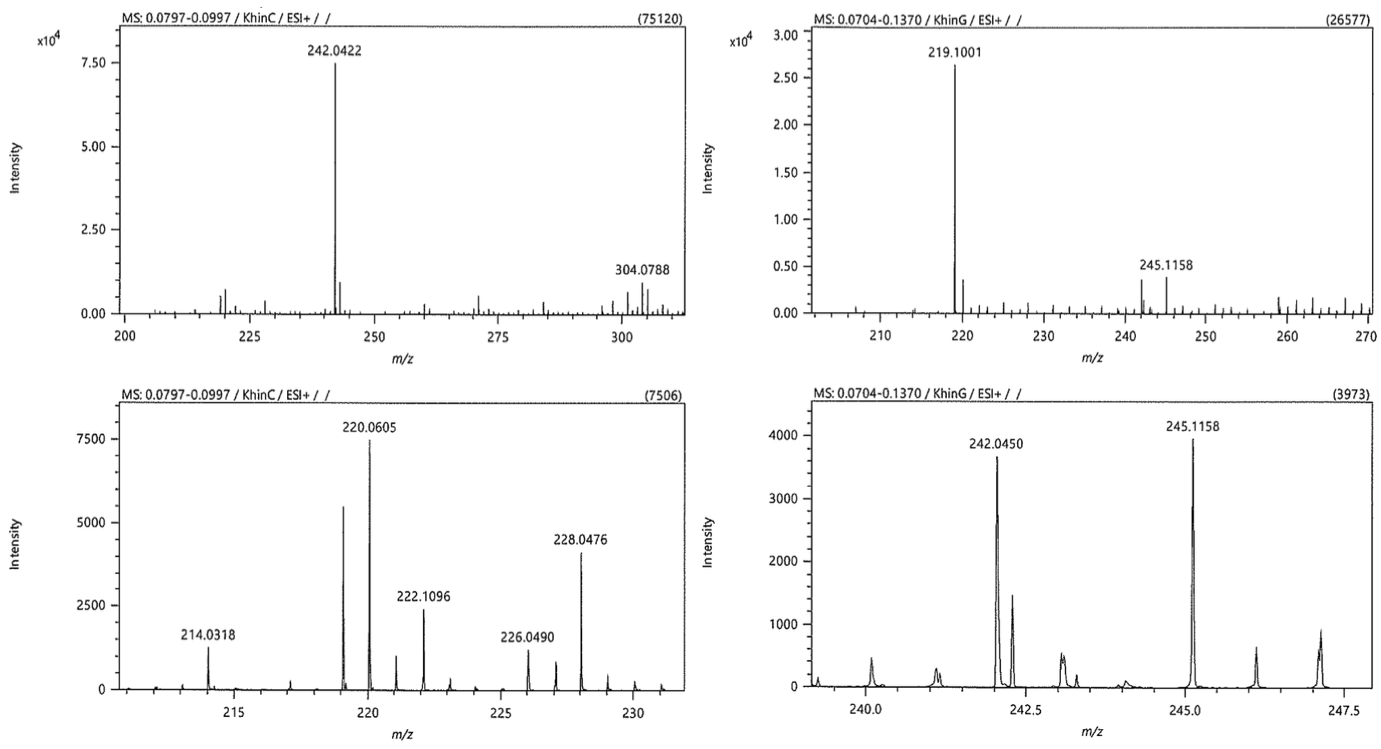


**Fig. S1. ESI/MS data of compound 1 of the active fraction (F10-1-2-3) of F-EtOAc extract.** The fractions of the F10-1-2-3 obtained by RP-HPLC were analyzed by ESI-mass spectrometry using the positive ion mode [M+Na]^+^.


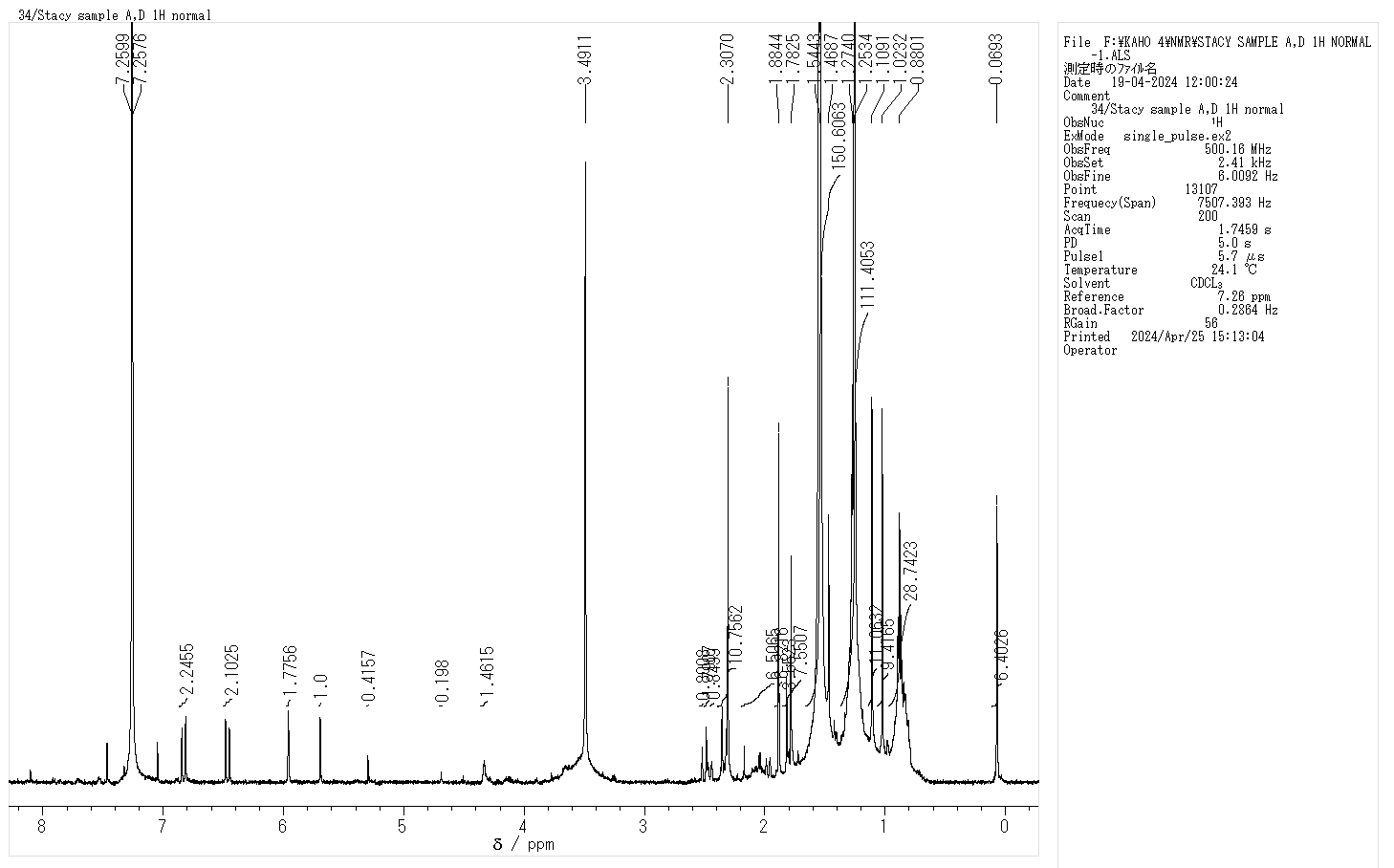


**Fig. S2. ^1^H-NMR spectrum of compound 1 of the active fraction (F10-1-2-3) of F-EtOAc extract (500 MHz, CDCl_3_).**


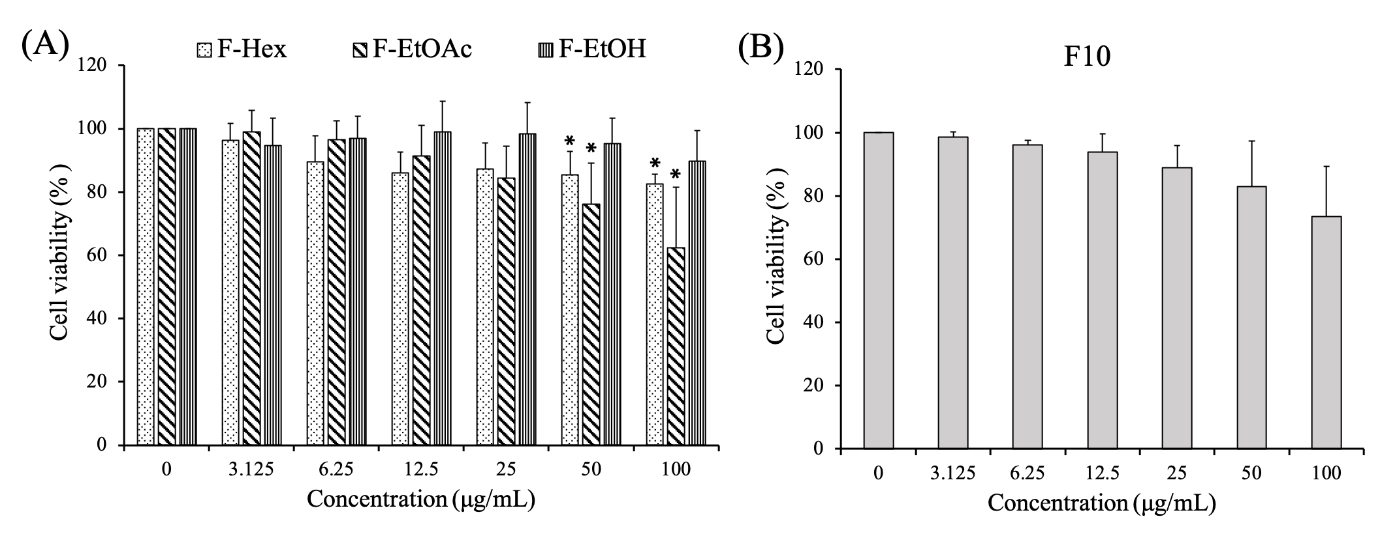


**Fig. S3. (A) Cytotoxicity of *G. pentaphyllum* hexane fractional extract (F-Hex), ethyl acetate fractional extract (F-EtOAc), and ethanol fractional extract (F-EtOH) on the PBMCs from five healthy volunteers represented as one group of healthy individuals. (B) Cytotoxicity of F10 on PBMCs from three healthy volunteers represented as one group of healthy individuals.** Data are presented as mean ± SD, based on average PBMCs data, with each experiment performed in triplicate. The asterisk (*) indicates a significant difference compared to 0 μg/mL; (**p* < 0.001, one-way ANOVA).


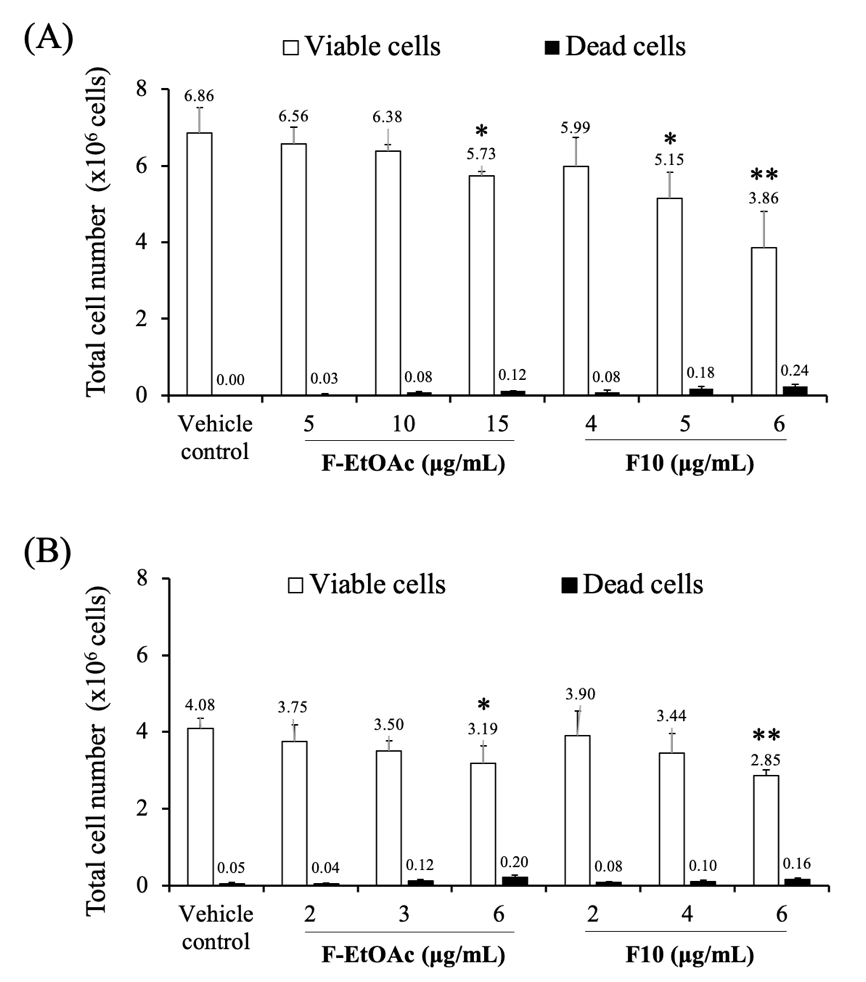


**Fig. S4. Effects of F-EtOAc and F10 on the total cell numbers in (A) EoL-1 and (B) MV4-11 leukemic cells, as determined by the trypan blue exclusion test.** Histograms show the mean of three independent experiments ± SD. The asterisk (*) denotes significant difference from vehicle control; (**p* < 0.05, ***p* <0.001, one-way ANOVA).

**Network Pharmacology**

**Screening of Common Targets of Dehydrovomifoliol and Luekemia**

The InChI and canonical SMILES profiles of dehydrovomifoliol were obtained from PubChem (<https://pubchem.ncbi.nlm.nih.gov/>), and was submitted to the Swiss Target Prediction database ([http://www.Swisstargetprediction.ch/](http://www.swisstargetprediction.ch/)) and the PharmMapper database (<https://lilab-ecust.cn/pharmmapper/index.html>) to determine dehydrovomifoliol–related targets, focusing specifically on *Homo sapiens* targets. The retrieved target genes were standardized to gene symbols using the UniProt database (<https://www.uniprot.org/>). Anti-leukemia targets were collected from the DisGeNET (<http://www.disgenet.org/>) and GeneCards (<https://www.genecards.org/>) databases.

To identify the common targets between dehydrovomifoliol and leukemia, the Venn diagram tool (<http://jvenn.toulouse.inra.fr/app/example.html>) and Microsoft Excel were used to remove duplicate entries. After intersecting dehydrovomifoliol–related targets with leukemia–related targets, 289 common targets were identified for further analyses. The targets were submitted to the STRING database (<https://string-db.org/>) (accessed on 30 October 2024) to explore the interactions. Protein–protein interaction (PPI) networks were created and visualized using Cytoscape 3.10.3. The final PPI network consisted of 289 nodes and 3,622 edges. As shown in Fig. S5, the top 10 targets with the highest degree values (indicating the most interactions) include TNF (degree = 135), AKT1 (degree = 132), ALB and IL6 (degree = 128), EGFR and HSP90AA1 (degree = 110), ESR1 (degree = 101), MAPK3 (degree = 98), and CASP3 and SRC (degree = 97).

**The Enrichment Analysis of Gene Ontology (GO) and Kyoto Encyclopedia of Genes and Genomes (KEGG) Pathway**

SR plot (<https://www.bioinformatics.com.cn/en>) was applied to evaluate the GO and KEGG enrichment.

**
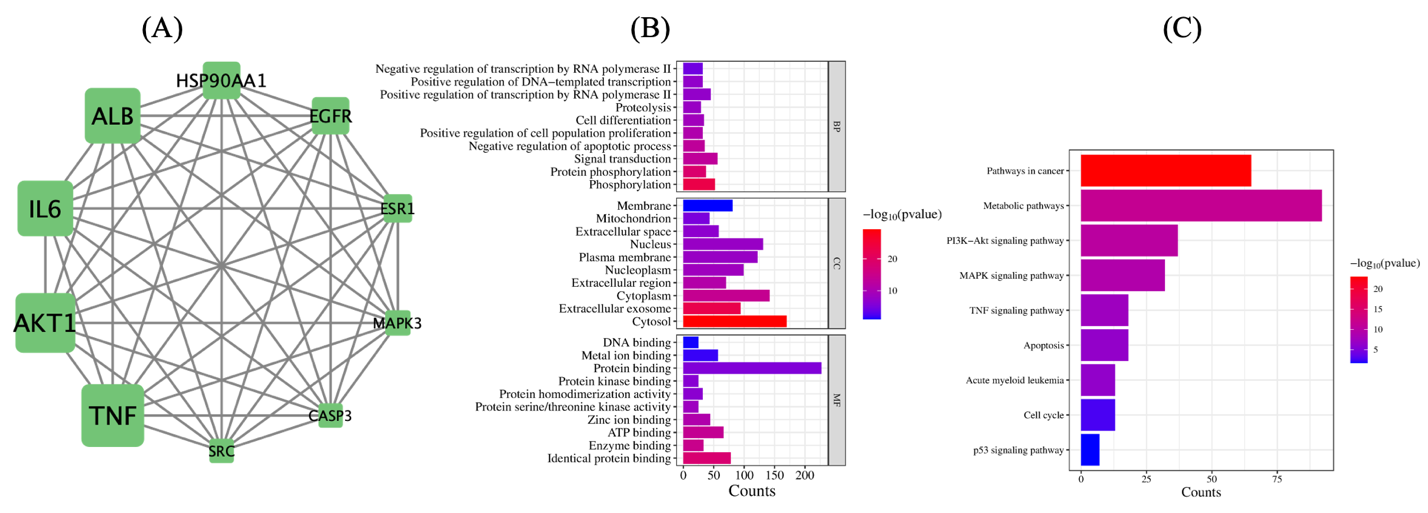
**

**Fig. S5. Network pharmacology analysis. (A) Protein–protein interactions (PPIs) network of the top 10 genes among leukemia**– **and dehydrovomifoliol**–**related targets.**Nodes represent proteins and edges represent protein–protein interactions. Node with larger font size and rectangular box indicates a higher degree within the network. **(B) Gene Ontology (GO) analysis of intersecting genes between dehydrovomifoliol and leukemia (top 10 targets by count rank), categorized into biological processes (BP), cellular components (CC), and molecular functions (MF). (C) Kyoto Encyclopedia of Genes and Genomes (KEGG) pathway analysis of core targets intersecting between dehydrovomifoliol and leukemia.**
