# Supplementary material for: Anti-leukemia activity of the ethyl acetate extract from Gynostemma pentaphyllum (Thunb.) leaf against FLT3-overexpressing AML cells and its phytochemical characterization
Source: BMC Complement Med Ther. 2025 May 13;25:172. doi: 10.1186/s12906-025-04903-0 (PMC12076849; doi:10.1186/s12906-025-04903-0)

**Anti-leukemia activity of the ethyl acetate extract from *Gynostemma pentaphyllum* (Thunb.) leaf against FLT3-overexpressing AML cells and its phytochemical characterization**

Khin Khin Gyi^1,2,3^, Songyot Anuchapreeda^1,4,5^, Nutjeera Intasai^1,4^, Montree Tungjai^6^, Siriporn Okonogi^5,7^, Arihiro Iwasaki^8^, Toyonobu Usuki^3*^, and Singkome Tima^1,4,5*^

*^1^Department of Medical Technology, Faculty of Associated Medical Sciences, Chiang Mai University, Chiang Mai 50200, Thailand*

*^2^Ph.D. Degree Program in Biomedical Sciences, Faculty of Associated Medical Sciences, Chiang Mai University, Under The CMU Presidential Scholarship, Chiang Mai, 50200, Thailand*

*^3^Department of Materials and Life Sciences, Faculty of Science and Technology, Sophia University, 7-1 Kioicho, Chiyoda-ku, Tokyo 102-8554, Japan*

*^4^Cancer Research Unit of Associated Medical Sciences (AMS CRU), Faculty of Associated Medical Sciences, Chiang Mai University, Chiang Mai 50200, Thailand*

*^5^Center of Excellence in Pharmaceutical Nanotechnology, Chiang Mai University, Chiang Mai 50200, Thailand*

*^6^Department of Radiologic Technology, Faculty of Associated Medical Sciences, Chiang Mai University, Chiang Mai 50200, Thailand*

*^7^Department of Pharmaceutical Sciences, Faculty of Pharmacy, Chiang Mai University, Chiang Mai, Thailand*

*^8^Department of Applied Chemistry, Faculty of Science and Engineering, Chuo University, 1-13-27 Kasuga, Bunkyo-ku, Tokyo 112-8551, Japan*

**Correspondence: singkome.tima@cmu.ac.th and t-usuki@sophia.ac.jp*

**Full uncropped Blot film images in the manuscript**

**Fig. 5A:** FLT3 protein expression


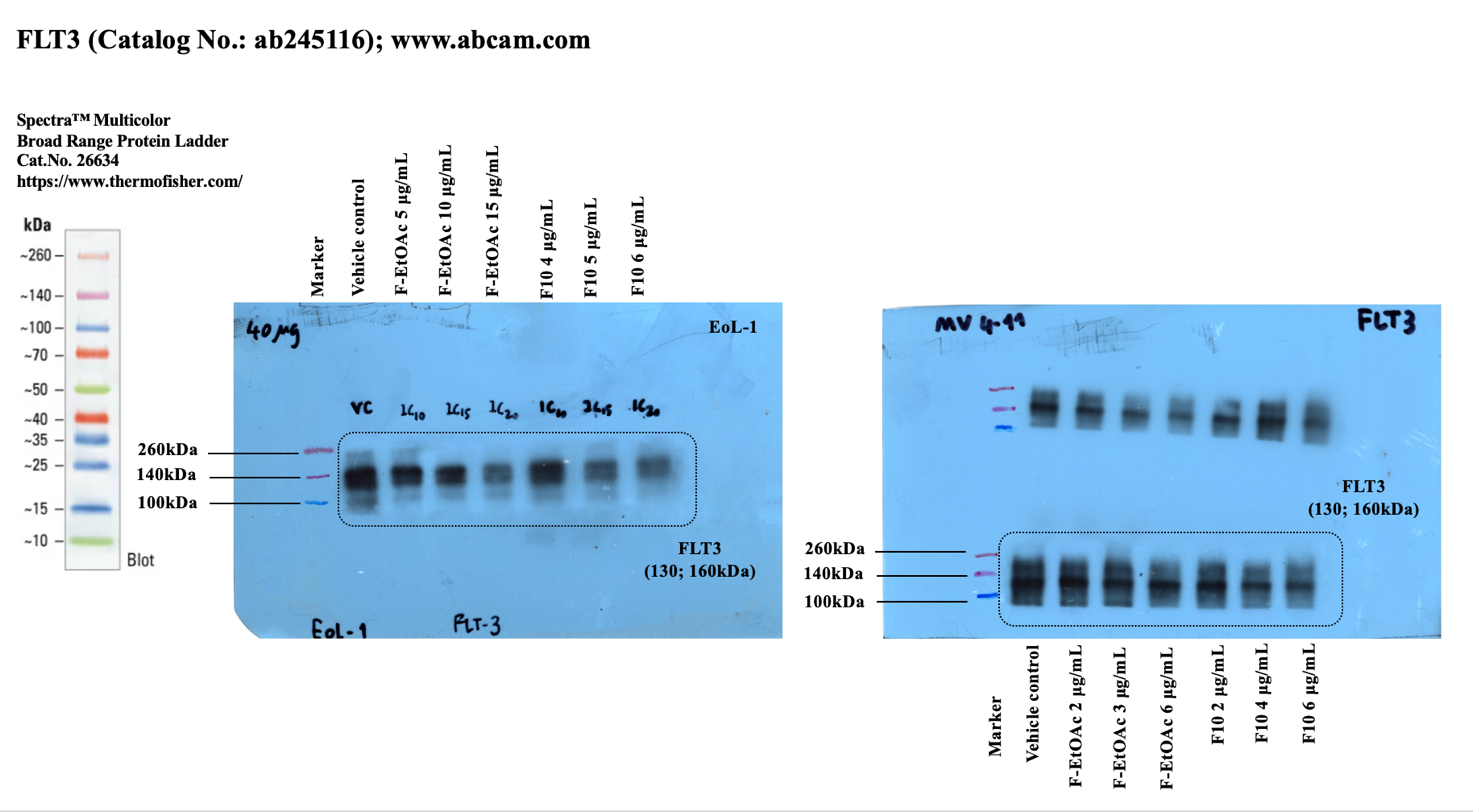


**Fig. 5B:** WT1 protein expression


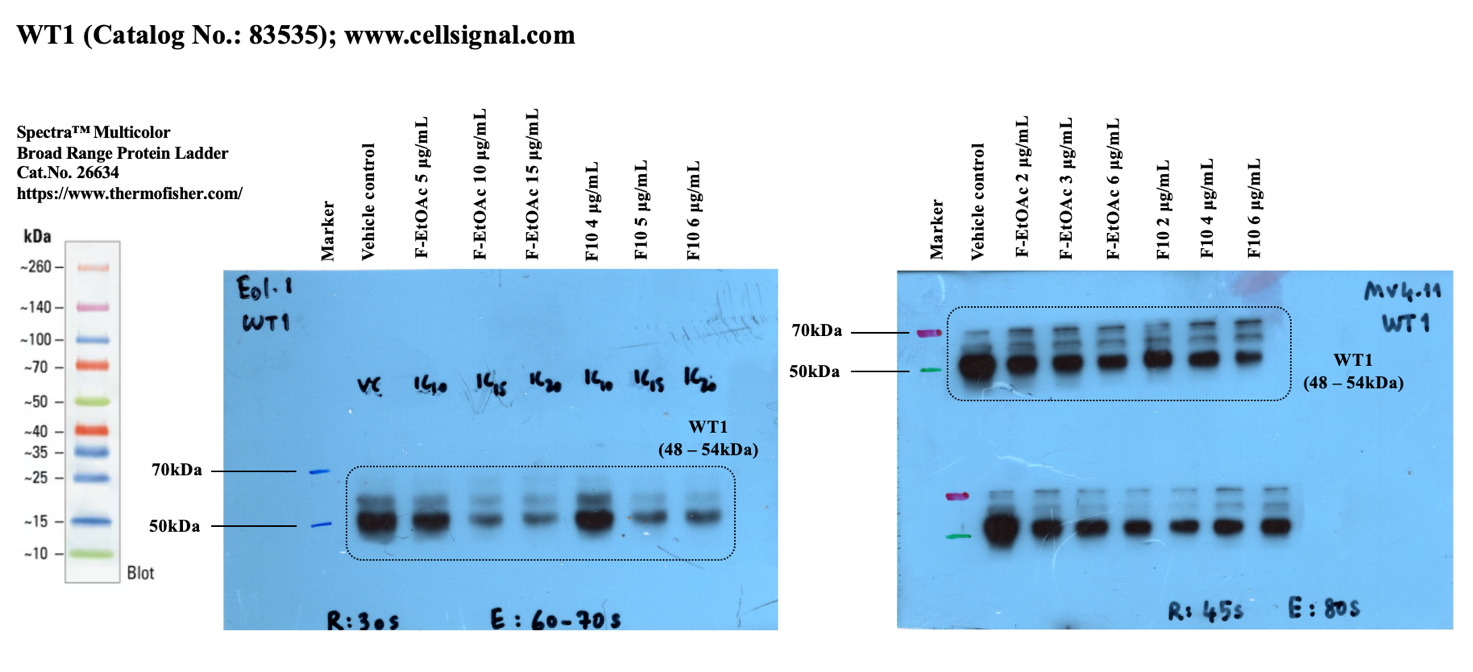


**Fig. 5:** GAPDH protein expression


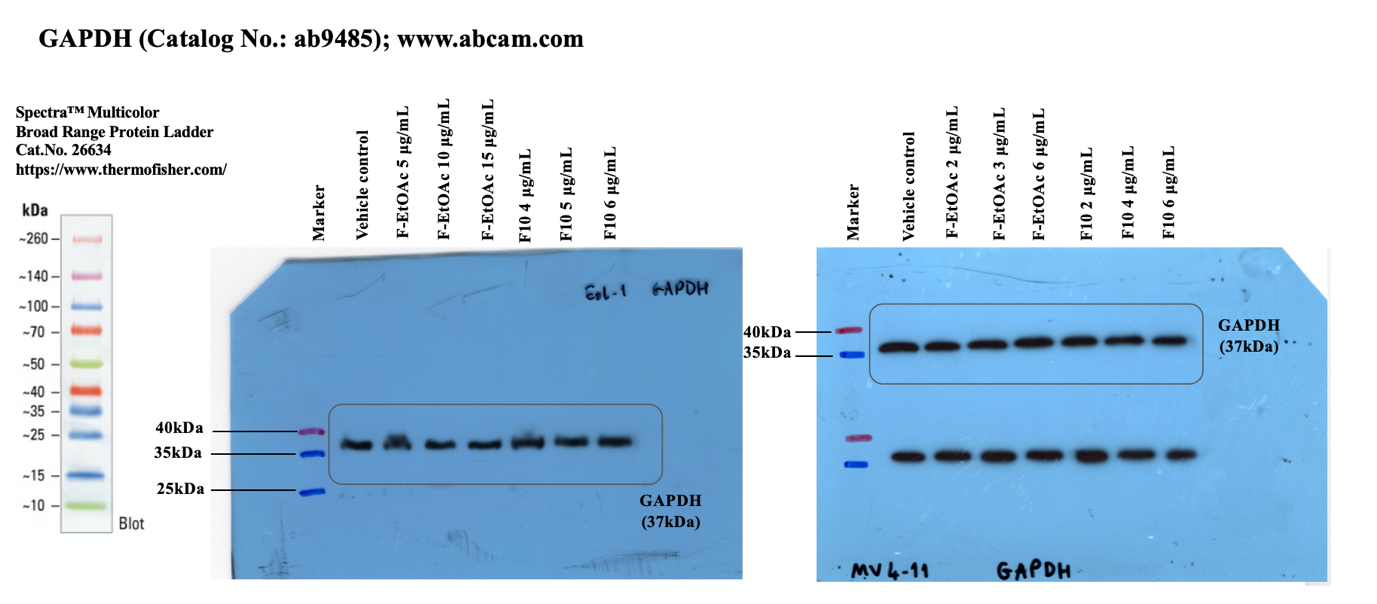


**Fig. 8:** p53 protein expression


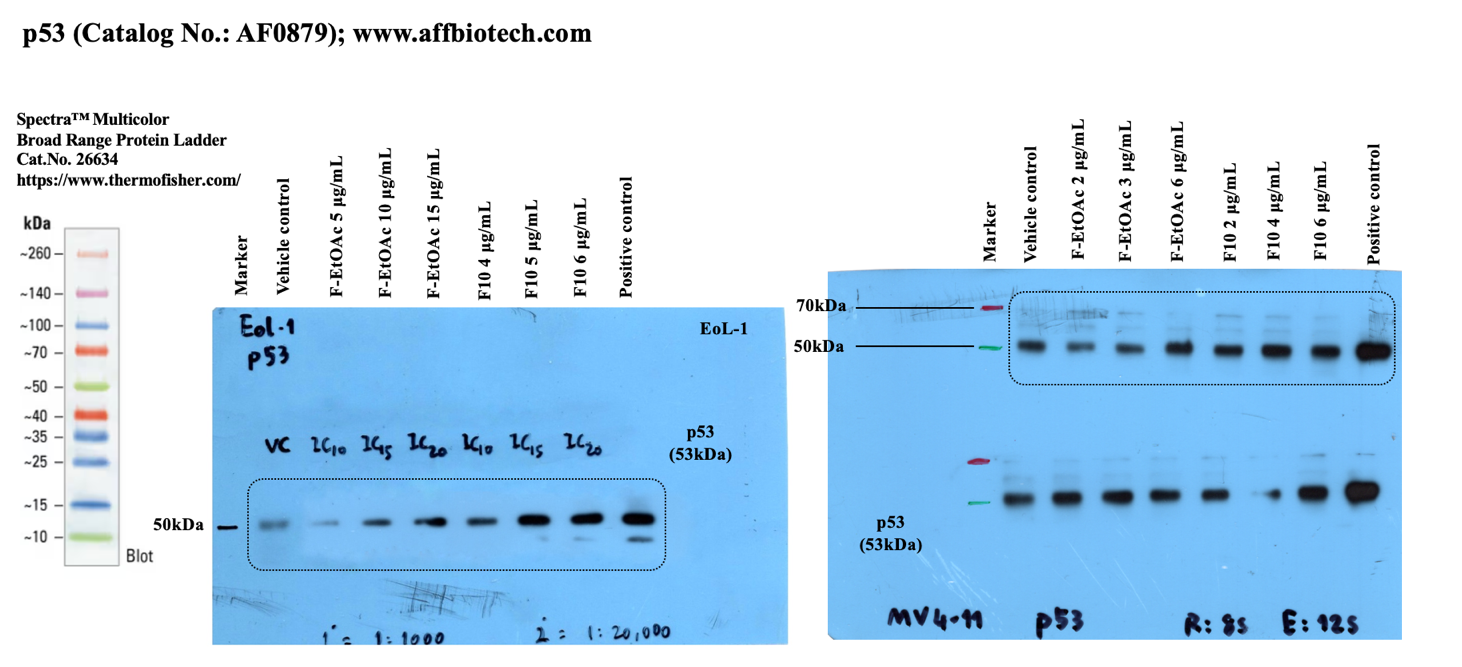


**Fig. 8:** Caspase-3 protein expression


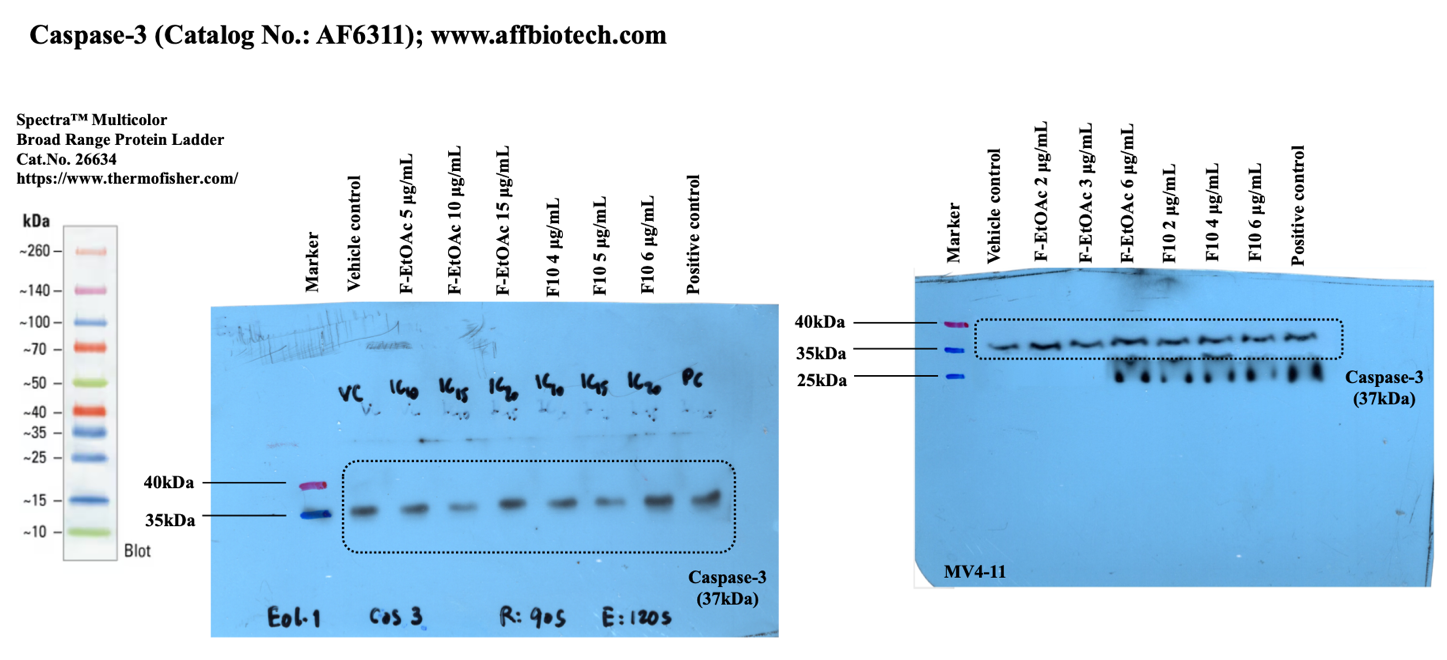


**Fig. 8:** Cleaved caspase-3 protein expression


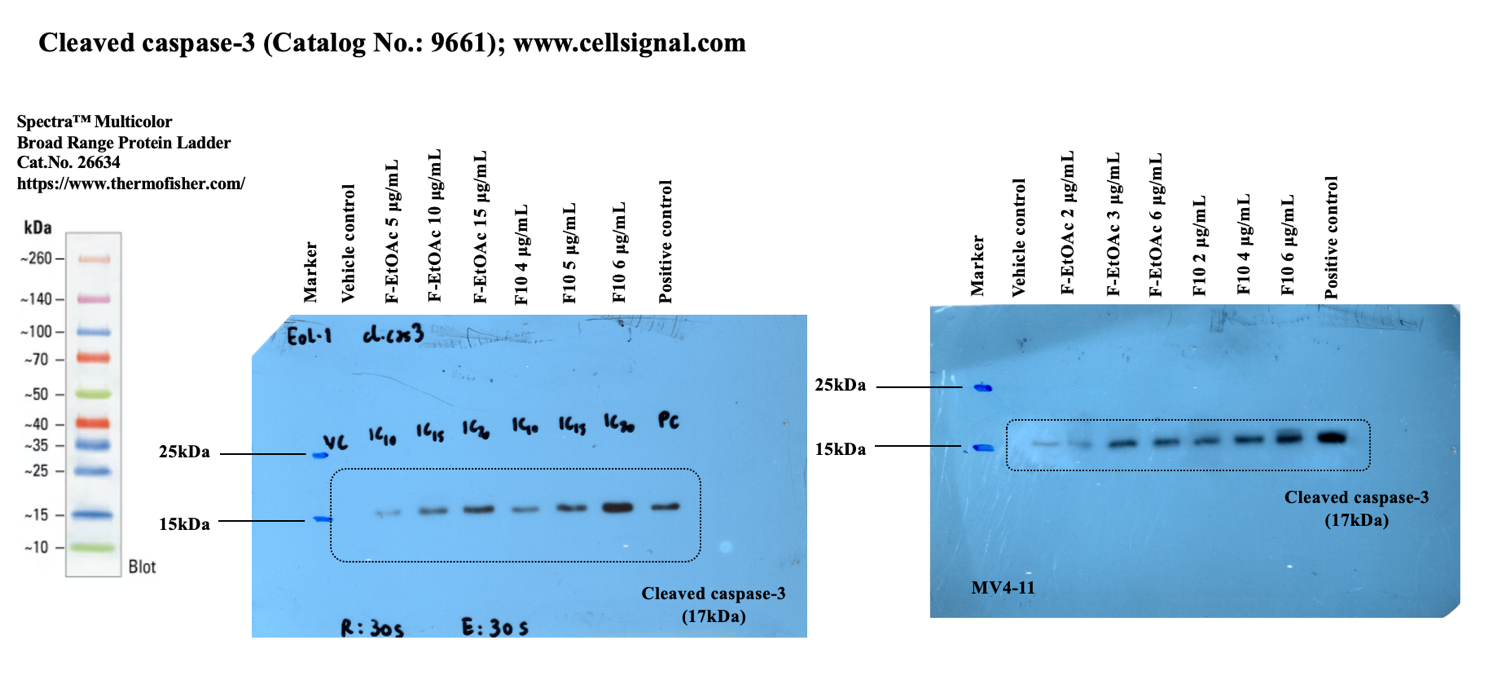


**Fig. 8:** GAPDH protein expression


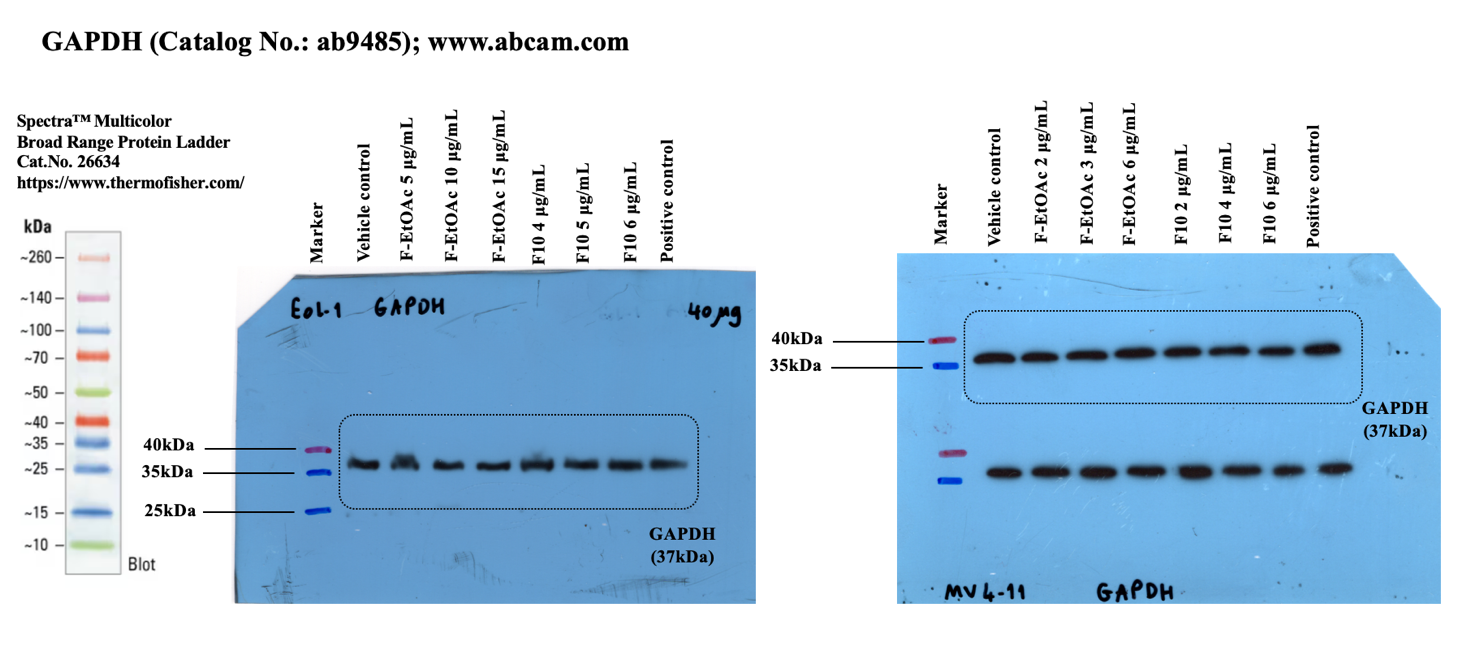

Supplement: Supplementary file 2 — Supplementary Material 2 [file 12906_2025_4903_MOESM2_ESM.docx]
